# Operando Study of Thermal Oxidation of Monolayer MoS_2_

**DOI:** 10.1002/advs.202002768

**Published:** 2021-03-01

**Authors:** Sangwook Park, Angel T. Garcia‐Esparza, Hadi Abroshan, Baxter Abraham, John Vinson, Alessandro Gallo, Dennis Nordlund, Joonsuk Park, Taeho Roy Kim, Lauren Vallez, Roberto Alonso‐Mori, Dimosthenis Sokaras, Xiaolin Zheng

**Affiliations:** ^1^ Department of Mechanical Engineering Stanford University Stanford CA 94305 USA; ^2^ Department of Mechanical Engineering Seoul National University Seoul 08826 South Korea; ^3^ Stanford Synchrotron Radiation Lightsource SLAC National Accelerator Laboratory 2575 Sand Hill Road Menlo Park CA 94025 USA; ^4^ SUNCAT Center for Interface Science and Catalysis SLAC National Accelerator Laboratory 2575 Sand Hill Road Menlo Park CA 94025 USA; ^5^ School of Chemistry and Biochemistry Georgia Institute of Technology Atlanta GA 30332 USA; ^6^ Linac Coherent Light Source SLAC National Accelerator Laboratory 2575 Sand Hill Road Menlo Park CA 94025 USA; ^7^ National Institute of Standards and Technology 100 Bureau Drive Gaithersburg MD 20899 USA; ^8^ Materials Science and Engineering Stanford University Stanford CA 94305 USA; ^9^ Stanford Nano Shared Facilities Stanford University Stanford CA 94305 USA

**Keywords:** 2D materials, monolayer molybdenum disulfide, operando oxidation, thermochemistry

## Abstract

Monolayer MoS_2_ is a promising semiconductor to overcome the physical dimension limits of microelectronic devices. Understanding the thermochemical stability of MoS_2_ is essential since these devices generate heat and are susceptible to oxidative environments. Herein, the promoting effect of molybdenum oxides (MoO*_x_*) particles on the thermal oxidation of MoS_2_ monolayers is shown by employing operando X‐ray absorption spectroscopy, ex situ scanning electron microscopy and X‐ray photoelectron spectroscopy. The study demonstrates that chemical vapor deposition‐grown MoS_2_ monolayers contain intrinsic MoO*_x_* and are quickly oxidized at 100 °C (3 vol% O_2_/He), in contrast to previously reported oxidation thresholds (e.g., 250 °C, *t* ≤ 1 h in the air). Otherwise, removing MoO*_x_* increases the thermal oxidation onset temperature of monolayer MoS_2_ to 300 °C. These results indicate that MoO*_x_* promote oxidation. An oxide‐free lattice is critical to the long‐term stability of monolayer MoS_2_ in state‐of‐the‐art 2D electronic, optical, and catalytic applications.

## Graphical abstract

Operando X‐ray absorption spectroscopy elucidates molybdenum oxide (MoO*_x_*) impurities’ effect on the thermochemical stability of monolayer MoS_2_. The thermal oxidation onset of as‐grown monolayer MoS_2_ is as low as 100 °C with MoO*_x_* present, overestimated by >150 °C in the literature (T ≥ 250 °C, t ≤ 1 h). Removal of MoO*_x_* increases the oxidation onset temperature to 300 °C.

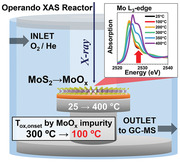

Monolayer molybdenum disulfide (MoS_2_) is an atomically thin semiconductor with a sub‐nanometer thickness (6.5–8 Å),^[^ ^1^, ^2^ ^]^ high electron mobility (≈200 cm^2^ V^−1^ s^−1^),^[^ ^1^ ^]^ a direct band gap (1.8–2.2 eV),^[^ ^1^, ^3^ ^]^ and high current on/off ratio (>10^8^).^[^ ^1^ ^]^ MoS_2_ monolayers^[^ ^4^ ^]^ can potentially be used for flexible electronics and push the physical dimension limits of microelectronics.^[^ ^4^, ^5^, ^6^ ^]^ Towards these visions, great progress has been made on the synthesis of wafer‐scale polycrystalline MoS_2_ monolayers,^[^ ^7^ ^]^ and large‐domain (≈500 µm) single‐crystalline MoS_2_ monolayers by chemical vapor deposition (CVD),^[^ ^2^ ^]^ and the wafer‐scale transfer and stacking of monolayer MoS_2_ for heterogeneous integrations.^[^ ^8^, ^9^ ^]^ In practice, microelectronics dissipate energy through Joule heating,^[^ ^10^ ^]^ and MoS_2_ monolayer transistors reach average operating temperatures of >150 °C with local hot spots of >250 °C.^[^ ^10^, ^11^ ^]^ Therefore, investigating the thermochemical stability of MoS_2_ above room temperature (RT) and under the device working temperature range is important.

Past studies on the thermal oxidation of MoS_2_ reported that the oxidation process is fast when the temperature is above ≈250 °C (e.g., 250 °C in the air for 1 h,^[^ ^12^ ^]^ 360 °C in the air for 5 min,^[^ ^13^ ^]^ and 380 °C in the air for 10 min; Table S1, Supporting Information).^[^ ^10^ ^]^ These findings were supported by ex situ observations of pits and crack formation, and identification of molybdenum oxides via atomic force microscopy (AFM),^[^ ^12^, ^13^, ^14^, ^15^, ^16^ ^]^ scanning electron microscopy (SEM),^[^ ^13^, ^15^, ^17^ ^]^ transmission electron microscopy (TEM),^[^ ^17^ ^]^ X‐ray photoelectron spectroscopy (XPS),^[^ ^12^, ^16^, ^17^ ^]^ nanomechanical means,^[^ ^16^ ^]^ and Raman spectroscopy.^[^ ^14^ ^]^ Such ex situ characterization methods, although very informative, may not detect subtle morphological and chemical compositional changes brought on by MoS_2_ oxidation. Importantly, the previous thermal oxidation studies assumed chemically pure MoS_2_ monolayers. However, many reported as‐grown monolayers frequently showed an XPS peak indicative of Mo^6+^ 3d_3/2_ from MoO_3_ at ≈236.5 eV.^[^ ^4^, ^18^, ^19^ ^]^ Even though molybdenum oxides (MoO*_x_*) are known to activate and transfer oxygen,^[^ ^20^, ^21^ ^]^ are defective,^[^ ^22^, ^23^ ^]^ and oxophilic,^[^ ^24^ ^]^ their effect on the oxidation of MoS_2_ has not been systematically investigated.

Herein, we characterize the thermal oxidation of MoS_2_ monolayers with and without MoO*_x_* at atmospheric pressure in the temperature range of 25–400 °C using a novel ultrasensitive operando X‐ray absorption spectroscopy (XAS). We report the first XAS measurement of an atomically thin monolayer MoS_2_ under dynamic environments (i.e., temperature ramping up to 400 °C, 3 vol% O_2_/He, 101.3 kPa, with X‐rays in the energy range of 2–3 keV) and it is complemented by ex situ XAS, SEM, and XPS. Such unique measurements of monolayer species were performed using our newly designed operando XAS reactor that enables an ultrasensitive electron yield (EY) detection mode under actual reactive environments;^[^ ^25^, ^26^, ^27^, ^28^, ^29^ ^]^ different to conventional fluorescence and transmission modes (see Table S2, Supporting Information, for details). Our study shows that as‐grown MoS_2_ monolayers via CVD contain trace amounts of oxygenated Mo^4+^ and Mo^6+^. Without removing the MoO*_x_*, the oxidation of MoS_2_ monolayer was activated starting as low as 100 °C even in a diluted oxidative atmosphere at ambient pressure (3 vol% O_2_ in inert gas). After removing MoO*_x_*, the oxidation onset temperature of MoS_2_ monolayers is increased to 300 °C. Our work shows that an oxide‐free and high‐purity monolayer MoS_2_ is critical to the enhanced thermochemical stability of MoS_2_.

To understand the effect of MoO*_x_* on the MoS_2_ oxidation, we prepared both as‐grown (oxide‐containing) and etched (oxide‐free) MoS_2_ monolayers with high quality supported on SiO_2_/Si wafers (see Experimental Section, Supporting Text and Figures S1–S4, Supporting Information, for details). The etched monolayer MoS_2_ was prepared by removing MoO*_x_* via an alkaline‐bath transfer treatment. We conducted electron microscopy, Raman, and XAS measurements to confirm that the etching step does not cause a structural and chemical change to the 2H‐MoS_2_ phase (**Figures** **1** and **2**; Figures S1, S2, and S4, Supporting Information). Ex situ SEM images in Figure 1 show the morphological evolution of the as‐grown and etched MoS_2_ monolayers. For all SEM images, the light background indicates the SiO_2_/Si support wafer (blue arrow), and the dark‐colored triangular shapes are monolayer MoS_2_ (red arrow) with tens of micrometers in size. Both the as‐grown and etched MoS_2_ samples appear to have unchanged global morphology even after oxidation at 350 °C, and both break up to small pieces at 400 °C (green arrow and dotted circles). The as‐grown and some oxidized MoS_2_ monolayers have lighter‐color nanoparticles arranged in lines (Figure S5, Supporting Information) as marked by orange arrows and guided lines in Figure 1. The nanoparticles are ascribed to molybdenum oxides (MoO*_x_*, vide infra). In comparison, those MoO*_x_* nanoparticles are not seen on the etched MoS_2_ (Figure 1k; Figure S6, Supporting Information), confirming the successful removal of oxide species by the alkaline treatment, consistent with the Mo Pourbaix diagram.^[^ ^30^ ^]^ Finally, for the etched monolayer MoS_2_, cracks and pits start to appear at 300 and 350 °C, which are indications of MoS_2_ oxidation (Figure 1n,o; and high magnification images in Figure 1r,s) and are also observed in the as‐grown MoS_2_ at the same annealing temperatures (Figure 1d,e; and high magnification images in Figure 1h,i). Overall, the SEM results suggest that the oxidation onset temperature of MoS_2_ monolayer is around 300 °C, regardless of the presence of MoO*_x_* nanoparticles, which is consistent with previous oxidation studies.^[^ ^12^, ^13^, ^14^ ^]^

**Figure 1:**
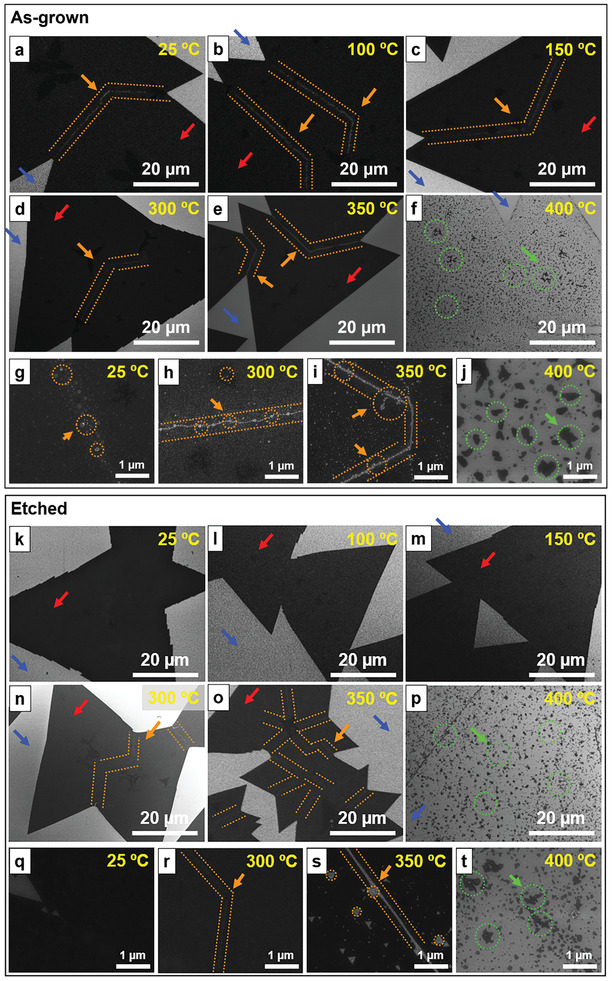
SEM images of the as‐grown and etched MoS_2_ monolayers before (25 °C) and after annealing (100–400 °C). a–j) The SEM images of the as‐grown MoS_2_ monolayers a,g) before annealing (25 °C) and after annealing at b) 100 °C, c) 150 °C, d,h) 300 °C, e,i) 350 °C, and f,j) 400 °C. k–t) The SEM images of the k,q) etched MoS_2_ monolayers before annealing (25 °C) and after annealing at l) 100 °C, m) 150 °C, n,r) 300 °C, o,s) 350 °C, and p,t) 400 °C. The light background is the SiO_2_/Si wafer (blue arrow) and monolayer MoS_2_ appears as a dark flake (red arrow). The bright nanoparticles (enclosed by orange dotted lines and pointed by arrows) are ascribed as MoO*_x_* nanoparticles. Both the f) as‐grown and p) etched MoS_2_ monolayers break into small pieces (green arrow and dotted circles) after being annealed at 400 °C. Ex situ SEM images were taken from different spots in different samples.

**Figure 2:**
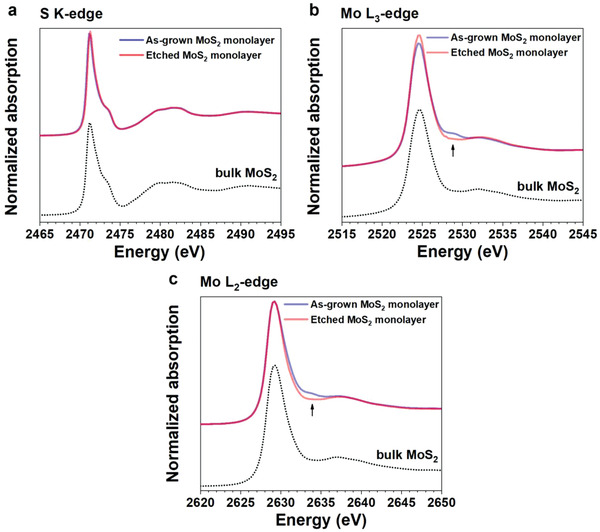
S K‐edge and Mo L_2,3_‐edges XANES spectra of the as‐grown and etched MoS_2_ monolayers with the bulk MoS_2_ reference. a) The S K‐edge, b) Mo L_3_‐edge, and c) Mo L_2_‐edge XANES spectra of the as‐grown (blue) and etched (red) MoS_2_ monolayers, and bulk MoS_2_ standard reference (black dot) are measured at room temperature (RT) under He atmosphere before oxidation. b,c) The additional shoulder peaks on the Mo L_3_ and L_2_‐edge spectra at b) 2528.6 eV and c) 2633.5 eV, respectively, (black arrows) from the as‐grown monolayer MoS_2_ indicate that the as‐grown monolayer MoS_2_ contains MoO*_x_*. The MoO*_x_* can be successfully removed from MoS_2_ monolayers by the alkaline bath treatment, which is confirmed through the removal of the additional shoulder peaks.

The operando XAS characterization reveals the details of the oxidation behavior differences for MoS_2_ monolayers with and without MoO*_x_*, which are not captured by SEM images. Our new operando XAS reactor equipped with EY detection mode enables, for the first‐time, XAS measurement of atomically thin MoS_2_ monolayers under reactive conditions (see the experimental scheme of the reactor in Figure S7 and Table S2, Supporting Information). The X‐ray beam spot size of 1 mm (vertical) × 3 mm (horizontal) is much larger than the single crystalline domain size of the monolayers (tens to around 100 µm). First, X‐ray absorption near edge structure (XANES) spectra of reference standards were obtained in the EY mode under He atmosphere under ambient pressure at RT using commercial bulk MoS_2_, MoO_2_, and MoO_3_ materials. The obtained spectra are consistent with the literature and our theoretical calculations (Figures S8 and S9, Supporting Information). Then, the S K‐edge and Mo L_3,2_‐edge XANES spectra of the as‐grown and etched MoS_2_ monolayers were measured under He atmosphere at RT. Though their spectra are similar to the bulk MoS_2_ material as shown in Figure 2, the as‐grown MoS_2_ monolayer shows additional shoulder peaks (black arrows) at 2528.6 eV in the Mo L_3_‐edge (Figure 2b) and 2633.5 eV in the Mo L_2_‐edge (Figure 2c), which indicates the presence of MoO*_x_*.

After confirmation of the existence of MoO*_x_* in the as‐grown MoS_2_ monolayers, operando XAS under the oxidative atmosphere was performed to follow the differences between the as‐grown and etched MoS_2_ monolayers with increasing temperature. The oxygen partial pressure used in the operando XAS electron yield reactor (3 vol% O_2_/He, 101.3 kPa) was identical to that used for the ex situ XAS, SEM, and XPS studies. The MoS_2_ monolayers were ramped to the desired temperature and held there for 30 min. Then, three consecutive overlapping XAS scans were recorded that show spectra reproducibility (Figure S10, Supporting Information), and the averaged spectra are used here. **Figure** **3** shows the operando XANES spectra of as‐grown (Figure 3a,c) and etched (Figure 3b,d) MoS_2_ monolayers under increasing temperatures (100–400 °C). The chemical evolutions of the as‐grown and etched MoS_2_ samples were monitored by following the S K‐edge (Figure 3a,b) and Mo L_3_‐edge (Figure 3c,d) XANES spectra.

Figure 3The operando S K‐edge and Mo L_3_‐edge XANES spectra of the as‐grown and etched MoS_2_ monolayers under dilute oxidative atmosphere (3 vol% O_2_/He) with increasing temperature (100–400 °C). a,b) The operando S K‐edge XANES spectra of the a) as‐grown and b) etched MoS_2_ monolayers are shown. The spectra of the commercial bulk MoS_2_ are included as references. The peak of S^6+^ is denoted at 2482.6 eV. c,d) The operando Mo L_3_‐edge XANES spectra of the c) as‐grown and d) etched MoS_2_ monolayers are shown. The prominent peak at 2528.6 eV generated by the gradual formation of MoO_3_ is marked with black arrows. The spectra of the commercial MoO_2_ and MoO_3_ are included as references. e,f) The difference spectra of the Mo L_3_‐edge XANES spectra of the e) as‐grown and f) etched MoS_2_ monolayers are shown. The spectra of the commercial MoO_2_ and MoO_3_ are included as references. g,h) The chemical compositions of g) as‐grown and h) etched MoS_2_ monolayers after oxidation at different temperatures are estimated from linear combination fitting analysis (LCFA).

**Figure:**
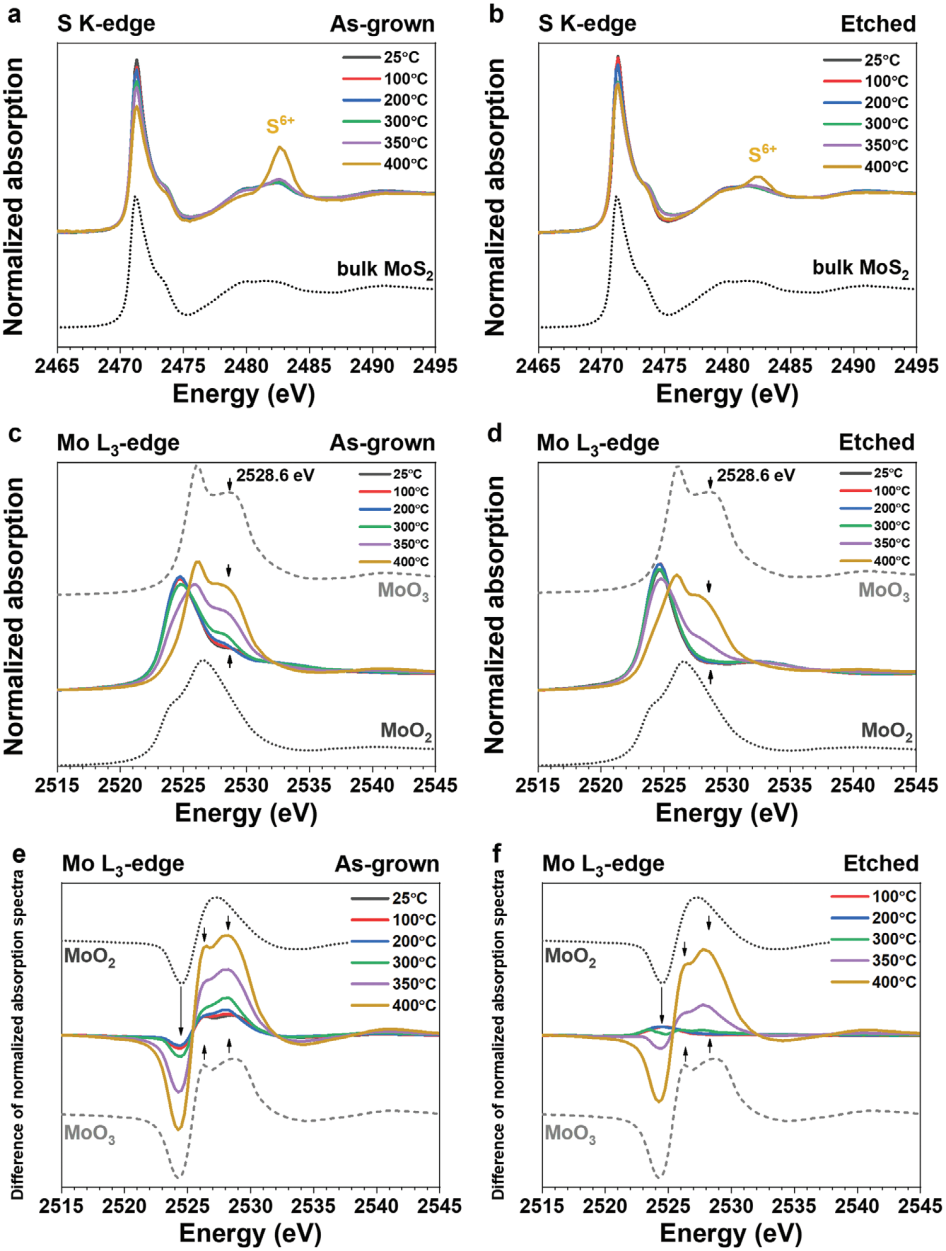

**Figure:**
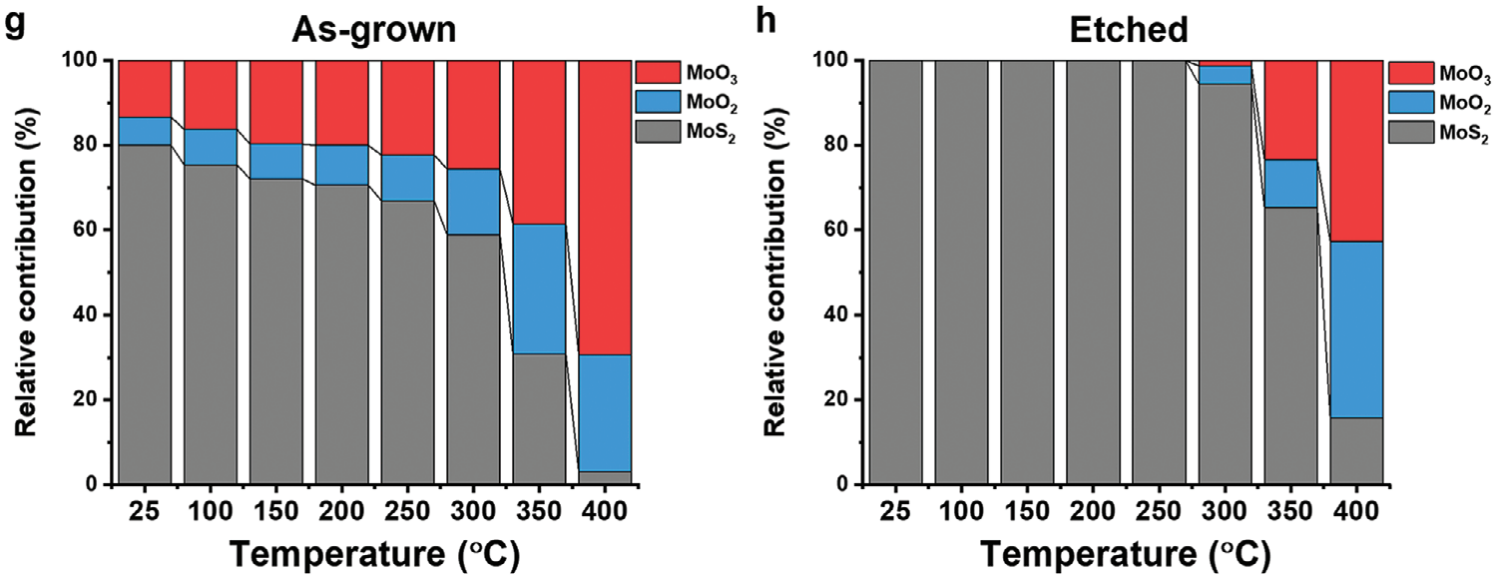

The evolution of the S K‐edge spectra is similar for the MoS_2_ monolayers with and without oxides. Both S K‐edge spectra features of the as‐grown and etched MoS_2_ monolayers match with the bulk MoS_2_ references for temperatures up to 350 °C, indicating that S atoms remain in the MoS_2_ chemical composition (Figure 3a,b). At 400 °C, both S K‐edge spectra show a new peak at 2482.6 eV, which is ascribed to S^6+^, likely in the form of sulfates.^[^ ^31^ ^]^ The difference in the intensity of the new S^6+^ peaks at 400 °C indicates that the as‐grown sample oxidizes more than the etched monolayer MoS_2_ (Figure 3a,b). The formation of sulfates at 400 °C coincides with the severe morphological changes observed via SEM images (Figure 1f,p).

On the other hand, the operando Mo L_3_‐edge XANES spectra show different evolution features between the as‐grown and etched MoS_2_ monolayers (Figure 3c,d). Those spectra were further subtracted by the spectra of the etched monolayer at 25 °C (i.e., pristine MoS_2_ without oxides) to obtain the difference spectra (Figures 3e,f), which highlight the changes of spectra feature. For the as‐grown MoS_2_ monolayer, the first negative broad feature at 2524.5 eV ascribed to a combination of Mo^4+^ and Mo^6+^ grows with increasing temperature up to 300 °C. Then, the negative feature shifts 0.3 eV to lower energy, resembling Mo^6+^ as in the MoO_3_ reference (Figure 3e). Likewise, the Mo L_3_‐edge spectra show a gradually increased Mo^6+^ peak at 2528.6 eV with increasing temperature (Figure 3c,e). Finally, the Mo L_3_‐edge white line is shifted from resembling MoS_2_ to MoO_3_ at 400 °C (Figure 3c,e). In comparison, for the etched sample, the Mo^4+^ and Mo^6+^ peaks ascribed to MoO_2_ and MoO_3_ become slightly apparent only at 300 °C, and the Mo L_3_‐edge white line is shifted from resembling MoS_2_ to MoO_3_ at 400 °C (Figure 3d,f). The Mo L_2_‐edge spectra show the same trends (Figure S11, Supporting Information). We cross‐checked the operando XAS results by conducting ex situ XAS of those MoS_2_ monolayers shown in Figure 1, and the results are consistent (Figure S12, Supporting Information). First‐principles simulations of XAS spectra with the OCEAN code^[^ ^32^, ^33^ ^]^ show that O‐substitution on S‐vacancies in MoS_2_ monolayers produces a sequential shift of the absorption edge with increasing oxygenation as observed in our experimental spectra (Figure 3c,e; Figure S13, Supporting Information). To summarize the Mo L_3_‐edge operando XANES results, oxygenated Mo^4+^ and Mo^6+^ species are formed starting at 100 °C in the as‐grown MoS_2_ monolayer sample, and those species increase with increasing temperature (likely in the form of MoO_2_ and MoO_3_). In contrast, features ascribed to oxides start to appear at 300 °C for the etched MoS_2_ monolayer sample. Furthermore, the as‐grown MoS_2_ monolayers are nearly completely oxidized at 400 °C, at which point, Mo^6+^ species (as in MoO_3_) dominate the spectra; however, the etched MoS_2_ monolayers still show a transition towards MoO_3_ formation at 400 °C likely resulting in the spectral combination of MoO_2_ and MoO_3_ signals.

To quantitatively investigate the trend of chemical composition changes of the as‐grown and etched MoS_2_ monolayers at different temperatures, the linear combination fitting analysis (LCFA) of the operando Mo L_3_‐edge XANES from Figure 3c,d was performed,^[^ ^34^ ^]^ and the results are presented in Figure 3g,h (see Tables S3 and S4, Supporting Information, for details). First, the chemical analysis supports the existence of MoO*_x_* in the as‐grown MoS_2_ monolayer, and on the contrary, the etched sample is composed of pure MoS_2_. Second, the amount of MoO*_x_* in the as‐grown sample is found to gradually increase with increasing temperature even at the low temperature of 100 °C, indicating the oxidation onset temperature (1% ± 1% MoO_2_ and 3% ± 1% MoO_3_ increase, and 5% ±1% MoS_2_ decrease). Third, the oxidation of the etched MoS_2_ monolayer starts at 300 °C. Complementarily, the normalized sulfur concentration acquired from the S K‐edge step as a function of temperature is shown in Figure S14, Supporting Information. The analysis of the edge step in the S K‐edge XANES spectra shows that both the as‐grown and etched MoS_2_ monolayers start losing significant amounts of S at around 350 °C, indicating that rapid oxidation happens. Both as‐grown and etched MoS_2_ monolayers undergo severe chemical transformations at 400 °C. The as‐grown monolayer MoS_2_ loses on average ≈23% more sulfur than the etched MoS_2_ monolayer under the same high temperatures and oxidation conditions at 350 and 400 °C. Overall, the differences between the samples in XANES and the chemical analysis suggest that the presence of MoO*_x_* lowers the onset temperature for fast thermal oxidation, promotes the oxidation reaction under the same temperature, and facilitates the sulfur loss in monolayer MoS_2_. Building upon published literature on the catalytic properties of oxides of molybdenum,^[^ ^20^, ^21^ ^]^ we propose that MoO*_x_* facilitates O_2_ dissociation ultimately lowering the onset oxidation temperature of as‐grown MoS_2_ monolayers down to 100 °C.

We further conducted complementary ex situ XPS at the O 1s, Mo 3d, and S 2p regions to confirm the impact of MoO*_x_* on the oxidation process of MoS_2_ monolayers. **Figure** **4**a,d shows the O 1s spectra of the as‐grown and etched MoS_2_ monolayers that are fitted with two singlets, respectively. The main peak at 533.4 eV for both samples is assigned to Si–O from the SiO_2_/Si substrate.^[^ ^35^ ^]^ The small peak at 531.4 eV corresponds to lattice Mo–O in oxide structures.^[^ ^12^, ^36^, ^37^ ^]^ For the as‐grown MoS_2_ monolayer, the O 1s peak intensity of Mo–O gradually increases from RT to 400 °C, so does the Mo^6+^ peaks at 236.5 eV (3d_3/2_) and 233.5 eV (3d_5/2_) in the Mo 3d region (Figure 4b).^[^ ^12^, ^38^, ^39^ ^]^ For the etched‐MoS_2_ layers, both the O 1s peak for Mo–O and the Mo^6+^ peaks are negligible below 300 °C (Figure 4d,e). The S 2p spectra show no significant differences throughout the oxidation process until 400 °C for both as‐grown and etched samples (Figure 4c,f). For both as‐grown and etched samples, the MoS_2_ monolayers seem to be nearly fully oxidized at 400 °C (Mo^6+^ ≈>90%, Figure S15, Supporting Information), as evidenced by the weak intensity of S 2p (Figure S16, Supporting Information; ten times magnified counts than in Figure 4f) and 2s peaks, and Mo^4+^ peaks at 233.3 eV (3d_3/2_) and 230.2 eV (3d_5/2_) in the Mo 3d region.^[^ ^12^, ^38^, ^39^ ^]^ The XPS results support the observations from the operando XANES spectra (Figure 3) in that MoO*_x_* activates the oxidation of MoS_2_ monolayer from the low temperature at 100 °C. Nevertheless, operando XANES shows advantages by providing quantitative and qualitative information of chemical changes with better sensitivity, such as the sulfur loss and chemical specificity with increasing temperature under reactive and dynamic environmental conditions. Ultimately, operando XAS provides the accurate chemical composition evolution of the reaction products under the actual working conditions of 2D devices.

**Figure 4:**
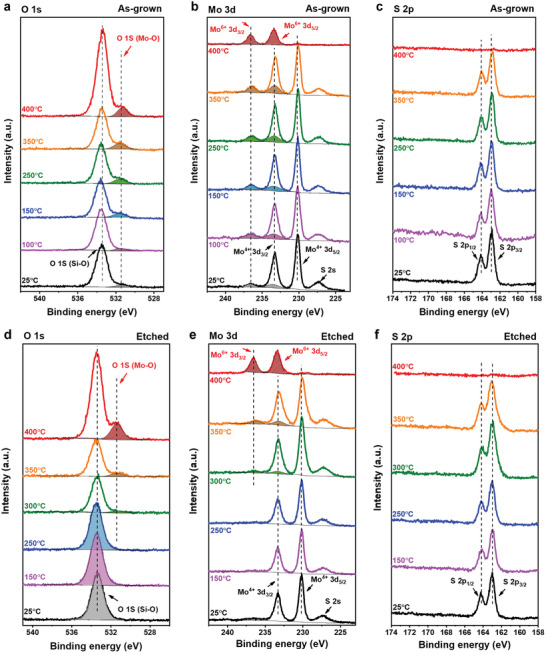
XPS spectra of O 1s, Mo 3d, and S 2p regions of the as‐grown and etched MoS_2_ monolayers before (25 °C) and after annealing (100–400 °C). a,d) XPS spectra of O 1s region of the a) as‐grown and d) etched MoS_2_ monolayers. The O 1s spectra are noted and partially fitted with the Mo–O (filled color with lines) and Si–O (filled color with dots) peaks. b,e) XPS spectra of Mo 3d region of the b) as‐grown and e) etched MoS_2_ monolayers. The Mo 3d spectra are noted and partially fitted with Mo^6+^ 3d_3/2_ and 3d_5/2_ peaks (filled color with lines). The other prominent peaks such as Mo^4+^ 3d_3/2_ and 3d_5/2_, and S 2s are denoted in each peak energy position. c,f) XPS spectra of the S 2p region of the c) as‐grown and f) etched MoS_2_ monolayers. The prominent peaks of S 2p_1/2_ and 2p_3/2_ are denoted in each peak energy position.

In summary, we reveal for the first time the low‐temperature thermal oxidation of monolayer MoS_2_ promoted by MoO*_x_*. Our pioneering ultrasensitive operando XAS study shows that the as‐grown MoS_2_ monolayers (via CVD) contain MoO_2_ and MoO_3_ impurities (i.e., MoO*_x_*) and that the amount of the oxides increases with increasing MoS_2_ oxidation even with exposures to diluted O_2_ at the low temperature of 100 °C (3 vol% O_2_/He, 101.3 kPa). In comparison, the etched MoS_2_ monolayers remain pristine until heating to 300 °C. The difference indicates that MoO*_x_* promotes the oxidation of MoS_2_ monolayers under the operation conditions of 2D devices. The available S atoms remain in a similar chemical and structural environment up to 350 °C, at which point MoS_2_ oxidizes to MoO_3_ and other volatile species. These results are supported by systematic ex situ XAS, XPS, and SEM characterizations. The results demonstrate that the oxide‐free condition is critical to the long‐term thermochemical stability of monolayer MoS_2_, which is expected to hold for other transition metal dichalcogenides monolayers with crucial implications for emerging scalable 2D electronic, optical, and catalytic applications.

## Conflict of Interest

The authors declare no conflict of interest.

## Supporting information

Supporting Information

## Data Availability

Research data are not shared.

## References

[1] B. Radisavljevic , A. Radenovic , J. Brivio , V. Giacometti , A. Kis , Nat. Nanotechnol. 2011, 6, 147. 21278752 10.1038/nnano.2010.279

[2] J. Lee , S. Pak , P. Giraud , Y. W. Lee , Y. Cho , J. Hong , A. R. Jang , H. S. Chung , W. K. Hong , H. Y. Jeong , H. S. Shin , L. G. Occhipinti , S. M. Morris , S. Cha , J. I. Sohn , J. M. Kim , Adv. Mater. 2017, 29, 1702206. 10.1002/adma.201702206 28692787

[3] H. M. Hill , A. F. Rigosi , K. T. Rim , G. W. Flynn , T. F. Heinz , Nano Lett. 2016, 16, 4831. 27298270 10.1021/acs.nanolett.6b01007

[4] V. K. Sangwan , H. S. Lee , H. Bergeron , I. Balla , M. E. Beck , K. S. Chen , M. C. Hersam , Nature 2018, 554, 500. 29469093 10.1038/nature25747

[5] D. Akinwande , N. Petrone , J. Hone , Nat. Commun. 2014, 5, 5678. 25517105 10.1038/ncomms6678

[6] M. Park , Y. J. Park , X. Chen , Y. K. Park , M. S. Kim , J. H. Ahn , Adv. Mater. 2016, 28, 2556. 26833813 10.1002/adma.201505124

[7] K. Kang , S. Xie , L. Huang , Y. Han , P. Y. Huang , K. F. Mak , C. J. Kim , D. Muller , J. Park , Nature 2015, 520, 656. 25925478 10.1038/nature14417

[8] J. Shim , S.‐H. Bae , W. Kong , D. Lee , K. Qiao , D. Nezich , Y. J. Park , R. Zhao , S. Sundaram , X. Li , H. Yeon , C. Choi , H. Kum , R. Yue , G. Zhou , Y. Ou , K. Lee , J. Moodera , X. Zhao , J.‐H. Ahn , C. Hinkle , A. Ougazzaden , J. Kim , Science 2018, 362, 665. 30309906 10.1126/science.aat8126

[9] K. Kang , K. H. Lee , Y. Han , H. Gao , S. Xie , D. A. Muller , J. Park , Nature 2017, 550, 229. 28953885 10.1038/nature23905

[10] E. Yalon , C. J. McClellan , K. K. H. Smithe , M. Munoz Rojo , R. L. Xu , S. V. Suryavanshi , A. J. Gabourie , C. M. Neumann , F. Xiong , A. B. Farimani , E. Pop , Nano Lett. 2017, 17, 3429. 28388845 10.1021/acs.nanolett.7b00252

[11] K. F. Mak , K. L. McGill , J. Park , P. L. McEuen , Science 2014, 344, 1489. 24970080 10.1126/science.1250140

[12] D. M. Sim , M. Kim , S. Yim , M.‐J. Choi , J. Choi , S. Yoo , Y. S. Jung , ACS Nano 2015, 9, 12115. 26503105 10.1021/acsnano.5b05173

[13] W. L. Spychalski , M. Pisarek , R. Szoszkiewicz , J. Phys. Chem. C 2017, 121, 26027.

[14] M. Yamamoto , T. L. Einstein , M. S. Fuhrer , W. G. Cullen , J. Phys. Chem. C 2013, 117, 25643.

[15] T. H. Ly , M.‐H. Chiu , M.‐Y. Li , J. Zhao , D. J. Perello , M. O. Cichocka , H. M. Oh , S. H. Chae , H. Y. Jeong , F. Yao , L.‐J. Li , Y. H. Lee , ACS Nano 2014, 8, 11401. 25343242 10.1021/nn504470q

[16] R. Szoszkiewicz , M. Rogala , P. Dabrowski , Materials 2020, 13, 3067. 10.3390/ma13143067 PMC7412186 32659964

[17] S. Park , S. Siahrostami , J. Park , A. H. B. Mostaghimi , T. R. Kim , L. Vallez , T. M. Gill , W. Park , K. E. Goodson , R. Sinclair , X. Zheng , Adv. Mater. 2020, 32, 2003020. 10.1002/adma.202003020 32743836

[18] X. Zhang , J. Grajal , J. L. Vazquez‐Roy , U. Radhakrishna , X. Wang , W. Chern , L. Zhou , Y. Lin , P. C. Shen , X. Ji , X. Ling , A. Zubair , Y. Zhang , H. Wang , M. Dubey , J. Kong , M. Dresselhaus , T. Palacios , Nature 2019, 566, 368. 30692651 10.1038/s41586-019-0892-1

[19] S. Bettis Homan , V. K. Sangwan , I. Balla , H. Bergeron , E. A. Weiss , M. C. Hersam , Nano Lett. 2017, 17, 164. 28073273 10.1021/acs.nanolett.6b03704

[20] R. H. Holm , Chem. Rev. 1987, 87, 1401.

[21] H. Arzoumanian , Coord. Chem. Rev. 1998, 178–180, 191.

[22] N. Zhang , X. Li , H. Ye , S. Chen , H. Ju , D. Liu , Y. Lin , W. Ye , C. Wang , Q. Xu , J. Zhu , L. Song , J. Jiang , Y. Xiong , J. Am. Chem. Soc. 2016, 138, 8928. 27351805 10.1021/jacs.6b04629

[23] A. Ruiz Puigdollers , P. Schlexer , S. Tosoni , G. Pacchioni , ACS Catal. 2017, 7, 6493.

[24] K. P. Kepp , Inorg. Chem. 2016, 55, 9461. 27580183 10.1021/acs.inorgchem.6b01702

[25] A. Erbil , I. G. Cargill , R. Frahm , R. F. Boehme , Phys. Rev. B: Condens. Matter Mater. Phys. 1988, 37, 2450. 10.1103/physrevb.37.2450 9944791

[26] W. T. Elam , J. P. Kirkland , R. A. Neiser , P. D. Wolf , Phys. Rev. B: Condens. Matter Mater. Phys. 1988, 38, 26. 10.1103/physrevb.38.26 9945159

[27] G. D. Moggridge , T. Rayment , R. M. Ormerod , M. A. Morris , R. M. Lambert , Nature 1992, 358, 658.

[28] R. B. Greegor , N. E. Pingitore Jr. , F. W. Lytle , Science 1997, 275, 1452. 9072808 10.1126/science.275.5305.1452

[29] J.‐J. Velasco‐Velez , C. H. Wu , T. A. Pascal , L. F. Wan , J. Guo , D. Prendergast , M. Salmeron , Science 2014, 346, 831. 25342657 10.1126/science.1259437

[30] A. G. Tyurin , Prot. Met. 2003, 39, 367.

[31] E. D. Risberg , F. Jalilehvand , B. O. Leung , L. G. Pettersson , M. Sandstrom , Dalton Trans. 2009, 3542. 19381417 10.1039/b819257j

[32] J. Vinson , J. J. Rehr , J. J. Kas , E. L. Shirley , Phys. Rev. B: Condens. Matter Mater. Phys. 2011, 83, 115106. 10.1103/PhysRevB.83.115106 PMC6508618 31080344

[33] K. Gilmore , J. Vinson , E. L. Shirley , D. Prendergast , C. D. Pemmaraju , J. J. Kas , F. D. Vila , J. J. Rehr , Comput. Phys. Commun. 2015, 197, 109.

[34] E. S. Jeong , C. I. Park , Z. Jin , I. H. Hwang , J. K. Son , M.‐Y. Kim , J.‐S. Choi , S.‐W. Han , Catal. Lett. 2015, 145, 971.

[35] H. A. Duarte , M. E. Sad , C. R. Apesteguía , Catal. Today 2019, 356, 399.

[36] J. Liu , S. Tang , Y. Lu , G. Cai , S. Liang , W. Wang , X. Chen , Energy Environ. Sci. 2013, 6, 2691.

[37] D. Guan , J. Li , X. Gao , C. Yuan , J. Power Sources 2014, 246, 305.

[38] J. Kibsgaard , Z. Chen , B. N. Reinecke , T. F. Jaramillo , Nat. Mater. 2012, 11, 963. 23042413 10.1038/nmat3439

[39] J. Gao , B. Li , J. Tan , P. Chow , T. M. Lu , N. Koratkar , ACS Nano 2016, 10, 2628. 26808328 10.1021/acsnano.5b07677

