# Supplementary material for: Operando Study of Thermal Oxidation of Monolayer MoS2
Source: Adv Sci (Weinh). 2021 Mar 1;8(9):2002768. doi: 10.1002/advs.202002768 (PMC8097340; doi:10.1002/advs.202002768)
Supplement: Supplementary file 1 — Supporting Information [file ADVS-8-2002768-s001.pdf]

© 2021 Wiley-VCH GmbH

ADVANCED SCIENCE Open Access

Supporting Information

for *Adv. Sci.,* DOI: 10.1002/advs.202002768

Operando Study of Thermal Oxidation of Monolayer MoS_2

Sangwook Park†, Angel T. Garcia-Esparza†, Hadi Abroshan, Baxter Abraham, John Vinson, Alessandro Gallo, Dennis Nordlund, Joonsuk Park, Taeho Roy Kim, Lauren Vallez, Roberto Alonso-Mori, Dimosthenis Sokaras, Xiaolin Zheng**

Supporting Information

Operando Study of Thermal Oxidation of Monolayer MoS_2

Sangwook Park[†], Angel T. Garcia-Esparza[†], Hadi Abroshan, Baxter Abraham, John Vinson, Alessandro Gallo, Dennis Nordlund, Joonsuk Park, Taeho Roy Kim, Lauren Vallez, Roberto Alonso-Mori, Dimosthenis Sokaras*, Xiaolin Zheng*

[†]These authors contributed equally to this work
*Corresponding Authors: dsokaras@slac.stanford.edu and xlzheng@stanford.edu

This PDF file includes:

Experimental Section
Supporting Text
Figures S1-S16
Tables S1-S4
References

Experimental Section

Synthesis of a polycrystalline 2H-MoS_2 monolayer. A homemade tube furnace system was used to synthesize polycrystalline 2H-MoS_2 monolayer on SiO_2/Si substrates by the chemical vapor deposition (CVD) method.[1, 2] Molybdenum trioxide (MoO_3) and sulfur (S) powders were loaded in alumina boats and the boats were placed inside of the quartz tube. The SiO_2/Si substrate was precleaned with acetone, isopropanol, and deionized water in a sonicator for 15 mins each and suspended on the MoO_3 loaded alumina boat. The boat containing MoO_3 with SiO_2/Si substrate was located in the center of the tube furnace where the annealing temperature was set at 750 ºC, and the boat containing S was placed upstream of the tube furnace where the annealing temperature was set to ≈ 200 ºC during the synthesis. Argon (Ar) gas was used as a carrier gas and the flow rate was controlled at 6 sccm in ambient pressure using a mass flow controller. The as-grown MoS_2 is in the 2H phase, confirmed by the Raman spectrum with a frequency difference of ≈ 18.7 cm^{-1} between in-plane and out-of-plane vibrational modes,[3] and transmission electron microscopy (TEM) with matched lattice constants of 2.7 Å and 1.6 Å to d-spacing of (100) and (110) planes respectively (JCPDS #37-1492)[4, 5] (Figure S1).

Preparation steps of as-grown and etched MoS_2 monolayer. The as-grown MoS_2 monolayer was prepared through the CVD synthesis of MoS_2 monolayer without any treatment. The as-grown MoS_2 monolayers contain impurity oxides as reported in the literature,[6, 7] and they are directly used in the experiments. The etched MoS_2 monolayers were prepared through the PMMA (poly(methyl methacrylate)) assisted wet-transfer method. The as-grown MoS_2 sample was spin-coated with PMMA and the prepared PMMA/MoS_2/SiO_2 was soaked in 1 M KOH for around 2 hrs. Then, the floating PMMA/MoS_2 film was fished out by a clean SiO_2/Si substrate. The PMMA

film was removed by soaking in acetone and chloroform for 1.5 hrs each. During this process, 1 M KOH etches oxides of molybdenum,[8] producing the etched MoS_2 (oxide-free) monolayers.

Ex-situ SEM, XAS, and XPS samples preparation of as-grown and etched MoS_2 monolayers. All ex-situ MoS_2 monolayer samples for SEM, XAS, and XPS were separately prepared in the tube furnace. The oxidation condition in the tube furnace was 3 vol % O_2/Ar (101.3 kPa) for 30 minutes at denoted temperatures for each spectrum. The temperature at 25 ºC represents the room temperature before annealing. Ex-situ SEM images and XPS spectra were taken in a high vacuum at room temperature. Ex-situ XAS spectra were taken under 100 vol % He in an ambient pressure at room temperature.

Materials characterization. The morphology of as-grown and etched MoS_2 monolayers was inspected by SEM (FEI Magellan 400 XHR and FEI Sirion operated at 5 kV). The oxidation state, chemical composition, and elemental ratio of as-grown and etched MoS_2 monolayers were measured by XPS (PHI Versaprobe 1 Scanning XPS Microprobe with monochromatized Al Kα excitation source) with calibration to the chemical state of C-C of adventitious C at 284.8 eV. Regarding adventitious physisorption/chemisorption on the produced MoS_2 monolayers, the O 1s, and S 2p XPS data shows no evidence of chemisorb nor physisorbed OH-species (Figure 4). The XPS peaks are well-defined and only ascribed to MoS_2 on SiO_2, as described in the main text. Specifically, the O 1s XPS spectra of the KOH-etched MoS_2 monolayers do not show OH-related peaks around 529.5 to 531.5 eV (Figure 4d, 25 °C spectra).[9, 10] Also, the S 2p XPS in Figure 4c shows a well-defined and narrow single S $2p_{3/2}$ and S $2p_{1/2}$ doublet ascribed to sulfur in MoS_2; which is not the case with S-O(H) species that generate an extra second doublet at higher energies

and/or increased FWHM.[9, 10] The confidence level for the ratio quantification accuracy of XPS is set to 10 % to reduce the misinterpretation attributed to the signal to noise ratio in the Mo 3d region. The vibrational modes of as-grown and etched MoS_2 monolayers were studied by Raman spectroscopy. The Raman spectroscopy was performed by WiTec 500 AFM/micro-Raman Scanning microscope and HORIBA Scientific LabRAM HR Evolution Spectrometer with a 532 nm excitation laser. The transmission electron microscope (TEM) image was taken by FEI Titan ETEM 80-300 at 80kV accelerating voltage. The XAS of as-grown and etched MoS_2 monolayers was performed at beamline 4-3 at the Stanford Synchrotron Radiation Lightsource (SSRL) at SLAC National Accelerator Laboratory. The XAS was conducted using a Si(111) monochromator that delivered a 1 mm(V) × 3 mm(H) X-ray beam at the sample position (90° with respect to the incident beam). The operando S K-edge and Mo $L_{3,2}$-edge XANES spectra were collected in the electron yield mode using a new gas-tight operando XAS reactor under flowing gas compositions controlled by mass flow controllers (105 sccm, 3 vol % O_2/He, 101.3 kPa, He 99.999 %). The temperature of the sample was accurately controlled in the reactor using a custom-made heating unit and K-type thermocouple to monitor the local temperature of the monolayers. The operando XAS data were collected online under dynamic environmental conditions of gas and temperature, and the XANES spectra were further processed using the ATHENA software which is part of the Demeter package, a graphical interface of the IFEFFIT code.[11] Simulations of the XAS spectra were performed via the OCEAN code[12, 13] that uses QUANTUM ESPRESSO[14] for ground-state density functional theory (DFT).

Statistical analysis. The operando and ex-situ XAS experiments were performed during three different beam time runs at SSRL-SLAC that consisted of three days of experiments, each. The

beam times were separated by months. For each XAS experimental run, fresh CVD-grown MoS_2 monolayers were synthesized. From each CVD batch, the wafer was divided into 10 mm x 5 mm pieces uniformly covered with MoS_2 monolayers. Half of the batch was used as as-grown samples, and the other half was subject to the etching and transfer protocol to generate MoO_x-free MoS_2 monolayer samples. Ultimately, the oxidation onset temperature and consistent spectra of as-grown and etched monolayer MoS_2 was measured for three different batches of samples, at different times of the year. The XAS analysis was done with ATHENA software (part of the DEMETER package v0.9.26). Linear combination fitting analysis (LCFA) reconstructs the samples' spectra using the model standards (i.e., etched monolayer MoS_2, MoO_2, and MoO_3). The goodness of fit is reported using the R-factor and the reduced chi-squared. As defined for LCFA of the XANES data ATHENA's author describes the R-factor as the mean square sum of the misfit at each data point. For the reduced chi-squared, due to the solitary nature of time sequence XAS measurements/scans, ATHENA assumes 1 as the value of measurement uncertainty, resulting in very small values for chi-squared. Hence, although single values cannot assert the goodness of the fit, it is possible to relatively compare successive fits as performed in this work.

Supporting Text

The state-of-the-art Operando XAS reactor. The apparatus herein described in Figure S7 enables operando XAS studies of atomically-thin and ultra-dilute samples. The objective is to obtain XAS data under varying and dynamic environmental conditions (gas mixture balanced with ultra-high purity He, ambient pressure, and from room temperature to 400 °C). For the operando XAS studies, the samples are subject to a heat-treatment under different gases at atmospheric pressure using our in-house developed reactor. A custom-made heating unit locally increases the temperature of the sample. An integrated K-type thermocouple is used to monitor the local temperature of the sample in real-time, while a temperature controller and a power supply are used to control the heating rate and final temperature of the substrate. Mass flow controllers are utilized to control the gas mixture and the total flow into the reactor. The gas mixture flows in and out of the gas-tight reactor exposing the sample to a very stable and constant gas volume and partial pressure per time unit. X-rays are illuminated from the front X-ray transparent window of the reactor and the XAS signal is recorded using a metallic collector which is kept on a constant potential difference to collect the electron yield from the samples (Table S1).[15]

Theoretical analysis of XANES spectra transition by the monolayer MoS_2 oxidation. Periodic DFT calculations were carried out for a MoS_2 monolayer without S-vacancies as shown in Figure S13. We also considered models where S-vacancies at different levels of concentration were generated, and the Mo atoms thus exposed are coordinated by O atoms, i.e., substitution of surface sulfur atoms by oxygen. To prepare the models, a 2H-MoS_2 monolayer with a supercell of lateral size (4×4) was constructed, and some of the S atoms of the model were replaced by O. For all models thus prepared, a vacuum region of 18 Å was used to decouple the periodic images. We

used the Vanderbilt method (GBRV pseudopotential library)[16] and RPBE functional.[17] The kinetic energy cutoff was chosen to be 500 eV and integration was carried out in the reciprocal space with (2 × 2 × 1) Monkhorst-Pack *k*-points. All calculations were spin-polarized and performed with the QUANTUM ESPRESSO package.[14] The obtained DFT-optimized structures (see Figure S13) were used to simulate their Mo L_3-edge spectra using the OCEAN package.[12, 13] This first-principles code generates X-ray absorption spectra based on both ground-state DFT and the numerical solution of the Bethe-Salpeter equation (BSE) within a basis of electron and hole states (and associated core-hole dielectric screening) provided by the DFT Kohn-Sham orbitals.[18, 19] The DFT electronic structure was calculated within the generalized gradient approximation using the QUANTUM ESPRESSO code.[14] The efficient numerical sampling of the Brillouin zone was enabled through the use of the Shirley interpolation scheme.[20] The DFT plane-wave basis cut-off energy was set to 100 Ry using the PBE functional within the generalized gradient approximation (GGA).[21] The *k*-points used in the OCEAN calculations were 3 × 3 × 1 for all supercell models. The real-space mesh for the BSE calculation was 12 × 12 × 24. The radius of the sphere in which the local basis is calculated was set to 3.0 Bohr to construct the PAW-style optimal projector functions (OPF). The screening of the core-hole interaction was done in real space using the random phase approximation up to a radius around the core of 6.0 Bohr.[22] The calculated Mo L_3-edge XANES spectra were numerically broadened via convolution with a Lorentzian with a half-width at half maximum (HWHM) of 0.5 eV.

Reasons for selecting the CVD method and alkaline-bath transfer treatment. We synthesized monolayer MoS_2 by CVD method and removed MoO_x using alkaline-bath transfer treatment due to their advantages. First, the mechanical exfoliation of monolayer MoS_2 contains MoO_x

impurities[7, 23] that are likely intrinsic impurities of the bulk MoS_2 crystals[24] or introduced during processing, such as thermal annealing or O_2 plasma to remove the tape residue during the mechanical exfoliation process.[23] Second, exfoliated MoS_2 samples are typically composed of both monolayers and multi-layers, which are good for applications where you can pinpoint the monolayers. On the contrary, our chemical vapor deposition (CVD) method grows large flakes and areas of MoS_2 monolayers,[1, 2, 25] which are suitable for XAS measurements as the X-ray beam illuminates broad parts of the sample. Third, the transfer and etching method is a common protocol to transfer CVD grown MoS_2 monolayers to other substrates.[26, 27] We did not observe significant structural changes of MoS_2 from the transfer process (Figure S4) other than removing the MoO_x impurities. The surface morphology of the etched monolayer MoS_2 does not show any significant changes compared with as-grown monolayer MoS_2 (Figure 1a vs. k). Based on the Raman spectra comparison (Figure S4) and TEM image (2H phase) with FFT analysis (Figure S1), there is no phase transition (e.g. 2H phase to 1T phase) nor structural changes after the etching process.

Plausible effects of MoO_xS_y impurity. Our XAS, XPS, and Raman data do not show the presence of MoO_xS_y in our CVD-grown MoS_2 monolayers. First, S K-edge and Mo $L_{3,2}$-edge XAS spectra in Figure 2 demonstrate a clear MoS_2 stoichiometry, structural motif, chemistry, and electronic structure at room temperature under an inert atmosphere. Second, the Mo 3d XPS spectra, specifically the Mo $3d_{5/2}$ and $3d_{3/2}$ peak ratio and narrow FWHM in Figure 4b do not show the mixed Mo state composition expected for MoO_xS_y compounds (i.e., the combination of Mo^{4+}, Mo^{5+}, and Mo^{6+}). Similarly, S 2p XPS in Figure 4c shows a well-defined single S $2p_{3/2}$ and S $2p_{1/2}$ doublet ascribed to sulfur in the MoS_2 environment. This is not the case when MoO_xS_y is present because the oxysulfides produce shoulders in the spectrum, change the peak ratio, and increase the

FWHM as reported in the literature.[28-34] Third, the chemical composition of MoO_xS_y was not captured by Raman spectroscopy (Figures S1 and S4), which would show a diverse mixture of peaks similar to MoS_2, MoO_2, Mo_4O_{11}, and MoO_3 (e.g. E' and A_1'). In practice, the MoO_xS_y compound is barely observed in literature except when the MoS_2 synthesis conditions are not completely optimized, such as when the MoO_3 precursor is too rich. From published studies,[34-36] MoO_xS_y is partially oxidized MoO_3 precursors during the sulfurization process under S deficient conditions. The morphology of MoO_xS_y appears as thick orthorhombic particles rather than triangular crystalline MoS_2 monolayer films. Furthermore, MoO_xS_y can be easily observed by an optical microscope due to its relatively large size of a few to tens of micrometers, which was not the case in this work. In conclusion, the existence and effect of MoO_xS_y are negligible.

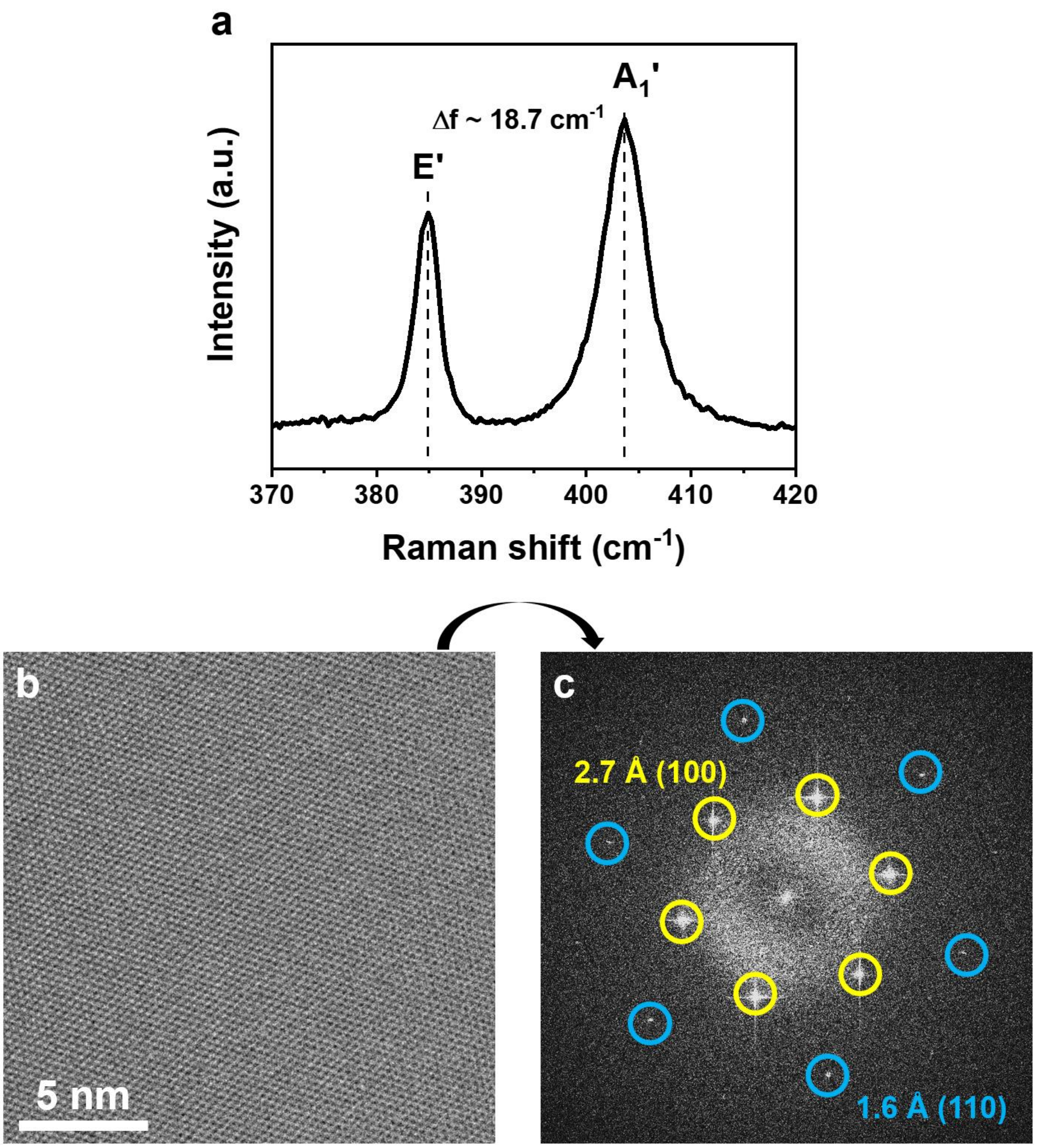

Figure S1. Raman spectra, TEM image, and the fast Fourier transform (FFT) image of 2H phase monolayer MoS_2. a) Raman spectrum of MoS_2 monolayers. The Raman spectrum shows in-plane (E') and out-of-plane (A_1') vibrational modes are 384.9 cm^{-1} and 403.6 cm^{-1} respectively with the frequency distance $\Delta f \approx 18.7$ cm^{-1} that indicates 2H phase MoS_2 monolayer.[3] b) TEM image of the monolayer MoS_2. c) FFT of TEM image in (b). (100) and (110) planes of monolayer 2H-MoS_2 are marked by yellow and light blue circles with a lattice distance of 2.7 Å and 1.6 Å respectively (JCPDS #37-1492).[4, 5]

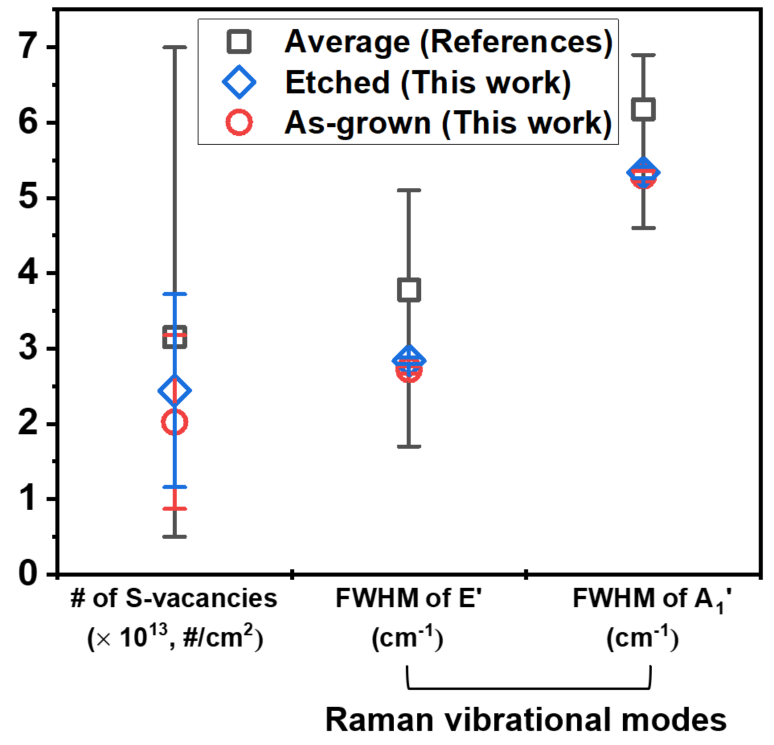

Figure S2. Material quality comparison of the as-grown and etched MoS_2 monolayers (this work) compared with literature references.[36-48] The concentration of S-vacancies and the full width half maximum (FWHM) of in-plane (E') and out of plane (A_1') vibrational modes of monolayer MoS_2 in Raman spectra are compared as representative parameters that show the quality of monolayer MoS_2.

The density of S-vacancies of our KOH etched MoS_2 monolayers (2.4×10^{13} #/cm^2) is comparable to our as-grown MoS_2 monolayers (2.0×10^{13} #/cm^2), indicating that the alkaline transfer/etching step has a minor impact on the defect density of monolayer MoS_2. Both S-vacancies concentrations are similar to the average of reported values (3.2×10^{13} #/cm^2)[37-43] within the uncertainty range (Figure S2). Second, both FWHM of as-grown (E': 2.7 cm^{-1} and A_1': 5.3 cm^{-1}) and etched (E': 2.8 cm^{-1} and A_1': 5.3 cm^{-1}) MoS_2 monolayers are smaller than the average reported values (E': 3.8 cm^{-1} and A_1': 6.2 cm^{-1}).[36, 43-48] Therefore, the quality of both as-grown and etched MoS_2 monolayers in our work is comparable or slightly better than the reported average MoS_2 monolayers.

The number of S-vacancies in the as-grown and etched MoS_2 monolayers is the measured S to Mo ratios from the Mo^{4+} 3d and S 2p regions by XPS (eight data points were averaged for the as-grown monolayers and six data points were averaged for the etched monolayers). The FWHM of E' and A1' of as-grown and etched MoS_2 monolayers are extracted by fitting with Gaussian functions in Figure S4 (R^2 = 0.99). The error bars in the average values from the references are the maximum and minimum literature values, and the number of S-vacancies in the as-grown and etched MoS_2 monolayers are standard errors. The error bars in the FWHM of E' and A1' of the as-grown and etched MoS_2 monolayers are from the Gaussian fitting of the measured spectra in Figure S4.

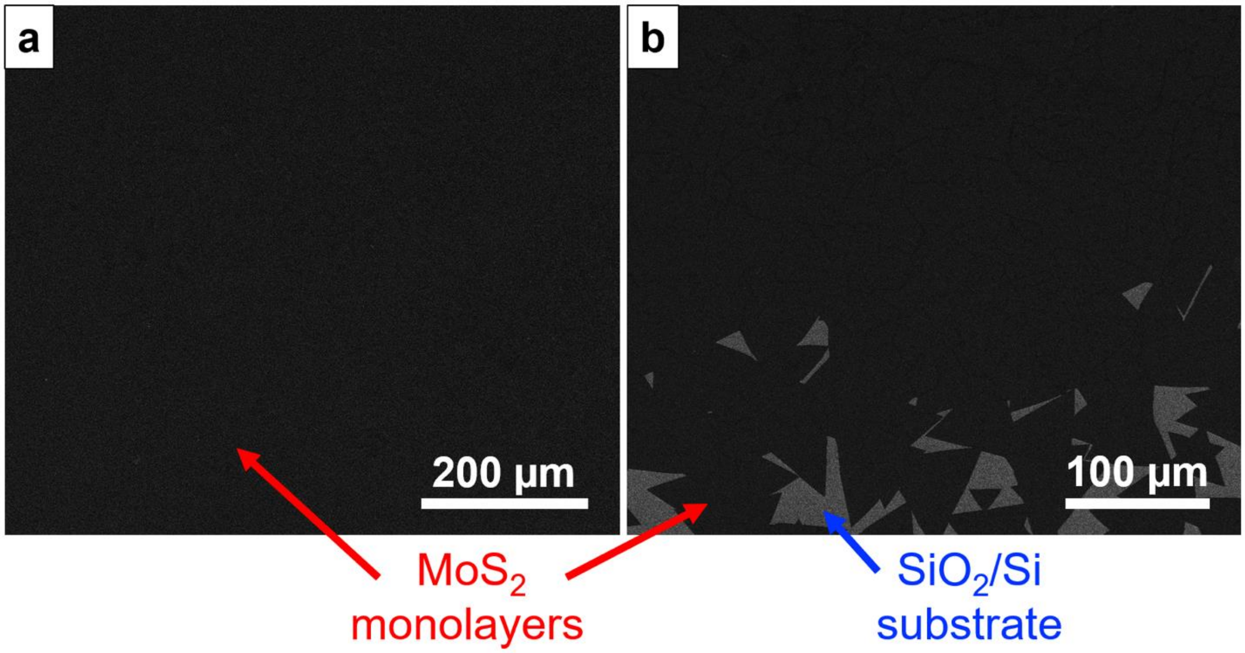

Figure S3. Representative SEM images of as-grown MoS_2 monolayers with relatively low magnifications. The SEM images show that our MoS_2 samples consist of continues single layer films (a) that are composed of crystalline domains with grain boundaries in the shape of flakes (b). The single crystalline domain (size of tens to around 100 µm) contains minimally exposed edges; hence, the edge sites to atomic bulk ratio is minimal. The effect of the edge sites in the oxidation process of monolayer MoS_2 should be negligible in the samples.

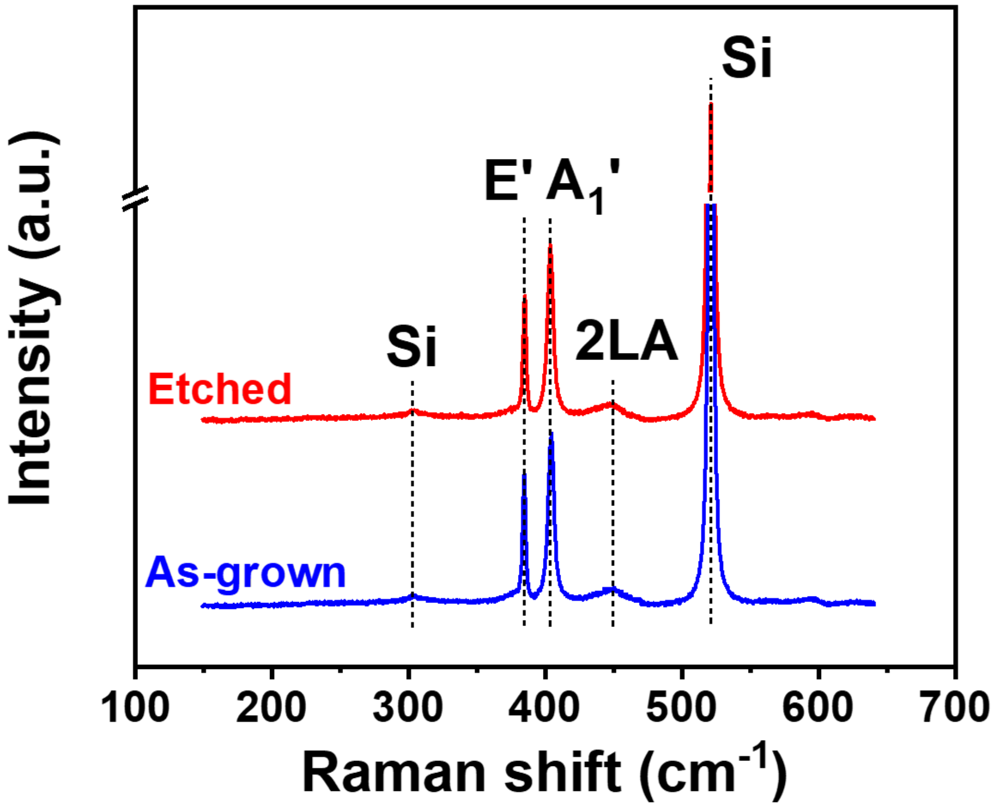

Figure S4. A broad range of Raman spectra of the as-grown and etched MoS_2 monolayers. Raman spectra of the as-grown and etched MoS_2 monolayers show the characteristic in-plane (E') and out of plane (A_1') vibrational modes at 385 cm^{-1} and 404 cm^{-1} and longitudinal acoustic vibrational mode at 450 cm^{-1} of the 2H phase monolayer MoS_2. Both Raman spectra do not contain the characteristic peaks of J_1 at 156 cm^{-1}, J_2 at 226 cm^{-1}, and J_3 at 330 cm^{-1} of 1T phase monolayer MoS_2.[49] Based on the Raman spectroscopy data, it is concluded that the alkaline etching process does not structurally affect the 2H phase of monolayer MoS_2. We could not detect MoO_3 Raman characteristic peaks which were observed from MoO_3 crystals in reference [50].

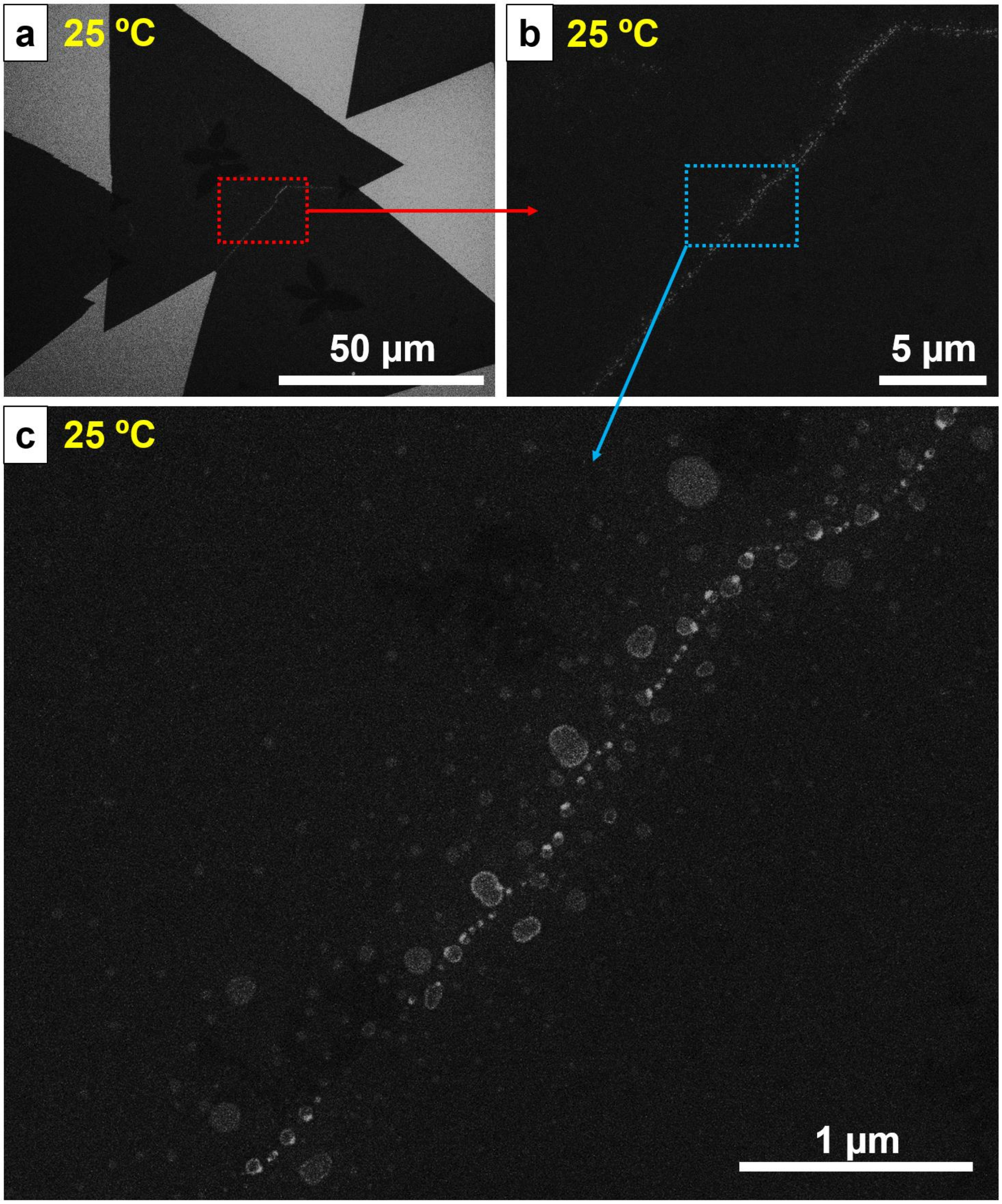

Figure S5. A sequence of SEM images of as-grown MoS_2 monolayers from low to high magnifications in the same spot. The crack-like bright line on the as-grown monolayer MoS_2 is a line cluster of the discontinuous individual MoO_x nanoparticles. A similar feature is shown from the as-grown MoS_2 samples annealed up to 250 °C and clear crack formation appears from the annealing temperature at 300 °C due to the oxidation of MoS_2 as shown in Figure 1.

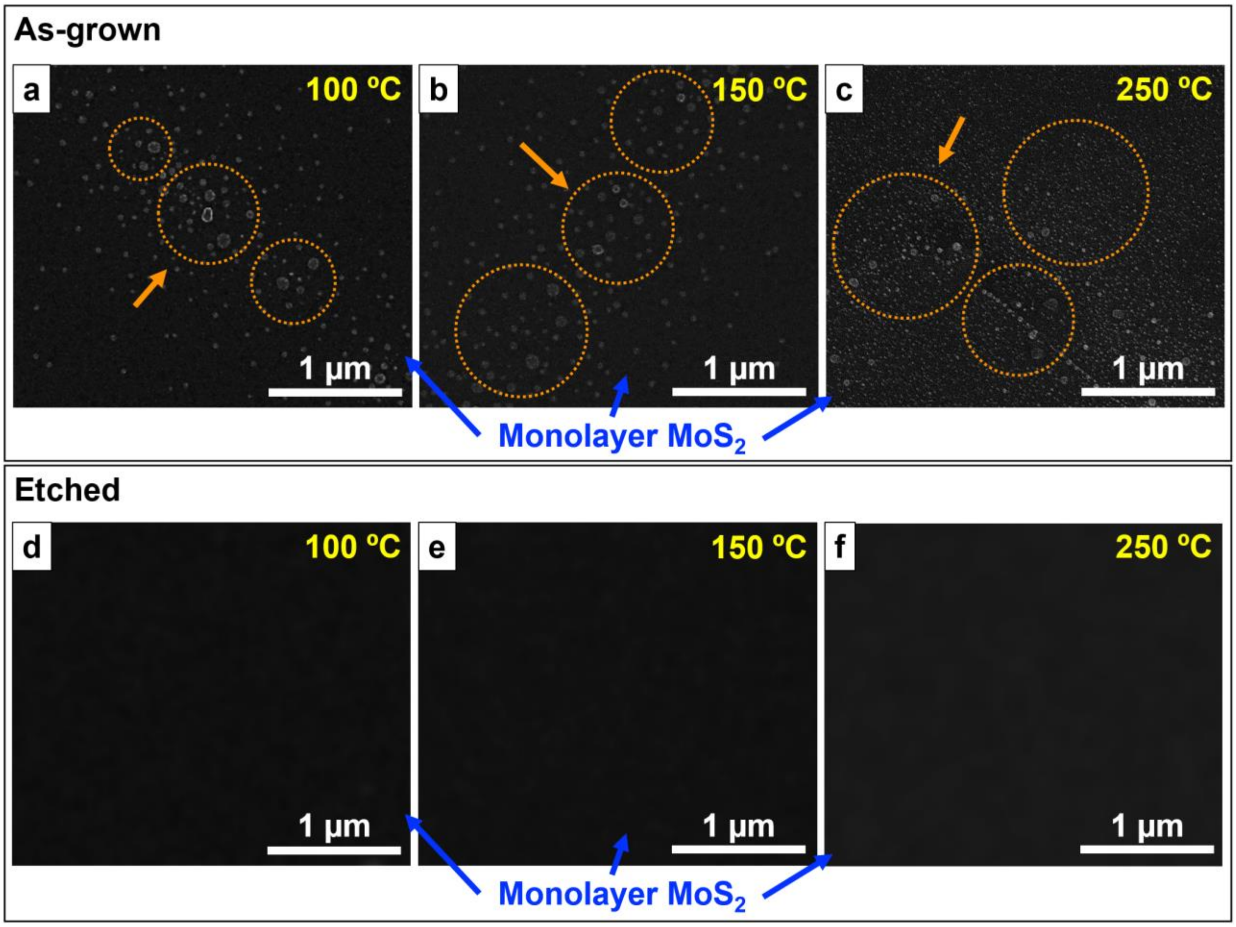

Figure S6. High-resolution SEM images of the as-grown and etched monolayer MoS_2 after annealing at 100 ºC, 150 ºC, and 250 ºC. a)-c) As-grown monolayer MoS_2 (black background pointed by blue arrows) annealed at 100 ºC (a), 150 ºC (b), and 250 ºC (c). The bright MoO_x nanoparticles are marked by orange dot circles and pointed by arrows. d)-f) Etched monolayer MoS_2 (black background pointed by blue arrows) annealed at 100 ºC (d), 150 ºC (e), and 250 ºC (f). As discussed in Figure 1k-t, the morphological changes and the generation of MoO_x nanoparticles are not observed at these annealing temperatures (100 ºC, 150 ºC, and 250 ºC) for the etched MoS_2 monolayers. All MoS_2 monolayer samples were separately prepared in the tube furnace and the SEM images were taken in a high vacuum at room temperature. The oxidation condition in the tube furnace was 3 vol % O_2/Ar under ambient pressure at the denoted temperatures (yellow) in each image for 30 mins.

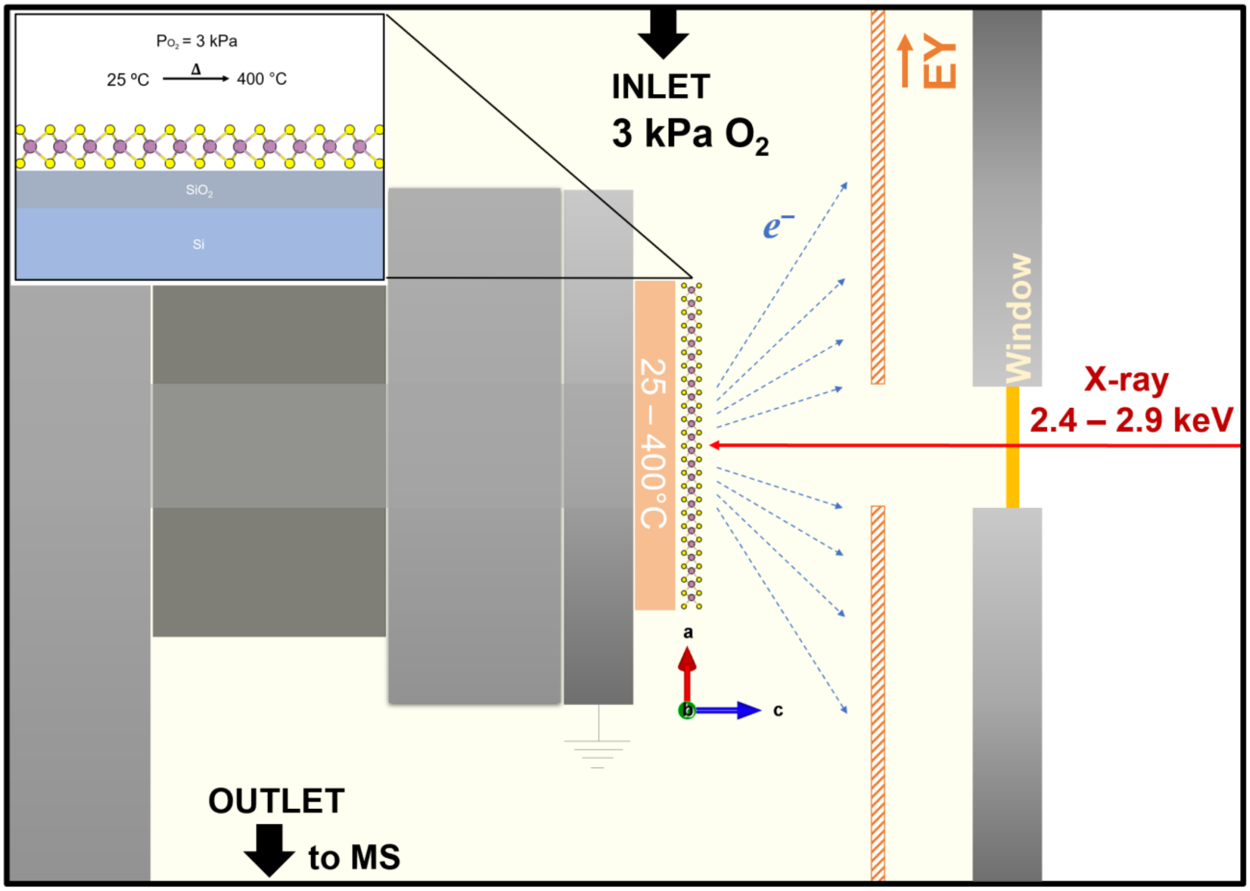

Figure S7. Schematic of the operando X-ray absorption spectroscopy (XAS) reactor with electron yield (EY) detection. The operando XAS reactor provides the capability of performing measurements under actual reactive environments (high temperature, ambient pressure, and diverse gas environments). The chamber of the reactor was designed to enable the measurement of atomically thin samples in the EY mode (monolayer and sub-monolayer concentration) by ensuring a very stable temperature and gas flow conditions which in turn allows very stable and almost noiseless measurements under realistic reactive environments.

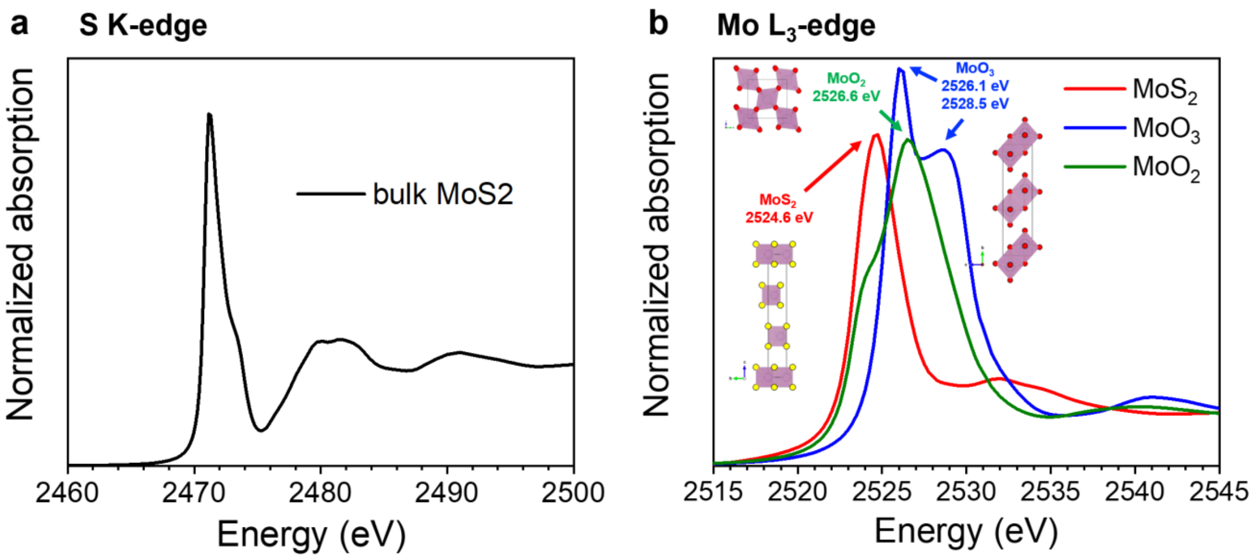

Figure S8. S K-edge and Mo L_3-edge XANES spectra of reference standard bulk samples of MoS_2, MoO_2, and MoO_3 obtained in the EY detection mode with our operando XAS reactor. a),b) The S K-edge of bulk MoS_2[51-54] (a) and Mo L_3-edge (b) XANES spectra of commercial bulk MoS_2,[53, 54] MoO_2,[55] and MoO_3[55, 56] as references (He atmosphere, ambient pressure, room temperature). Powdered reference samples were obtained from commercial sources and used as received to fabricate pellets of MoS_2 (98%, Aldrich, CAS 1317-33-5), MoO_2 (99%, Aldrich, CAS 18868-43-4), and MoO_3 (99.9995%, Alfa Aesar, CAS 1313-27-5).

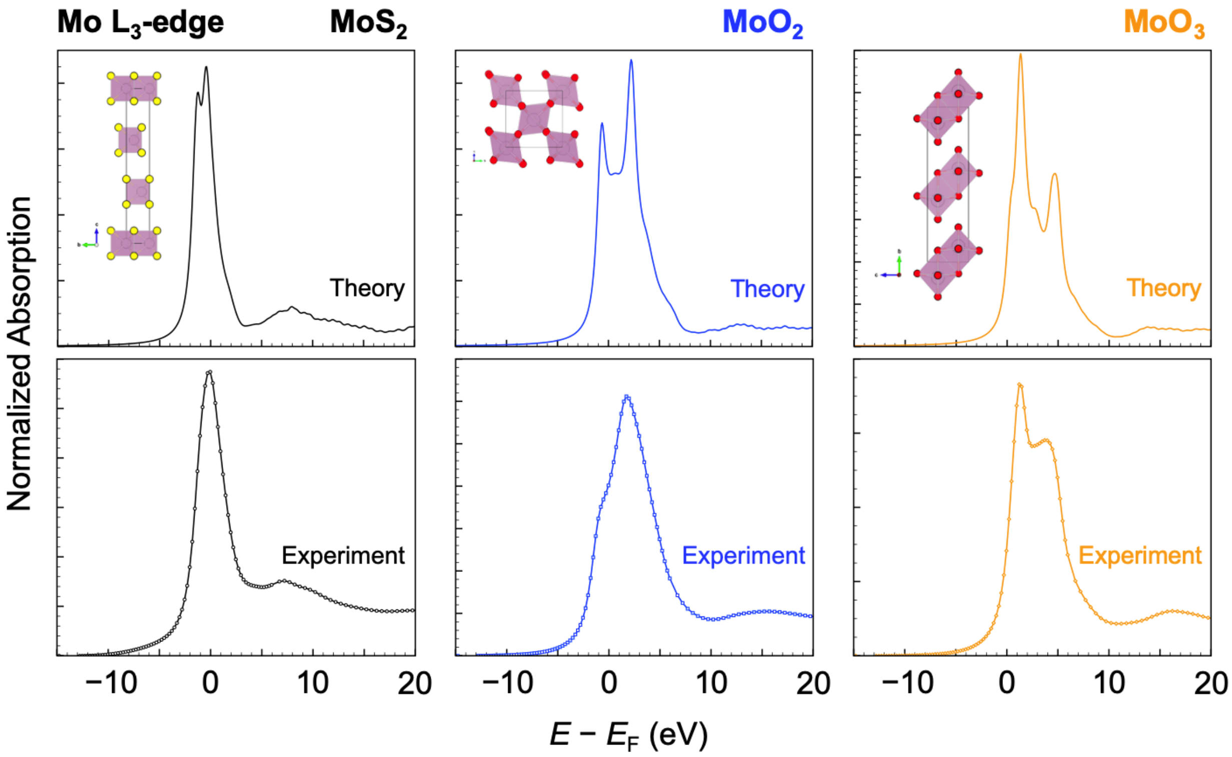

Figure S9. Mo L_3-edge XANES spectra of bulk MoS_2, MoO_2, and MoO_3 standards (bottom) and the first principles theoretical calculations of the spectra using their corresponding crystallographic structures (top). The theoretical spectra were calculated with the OCEAN code using crystal structures obtained from the Inorganic Crystal Structure Database (ICSD). Molybdenum atom in grey, oxygen in red and sulfur in yellow.

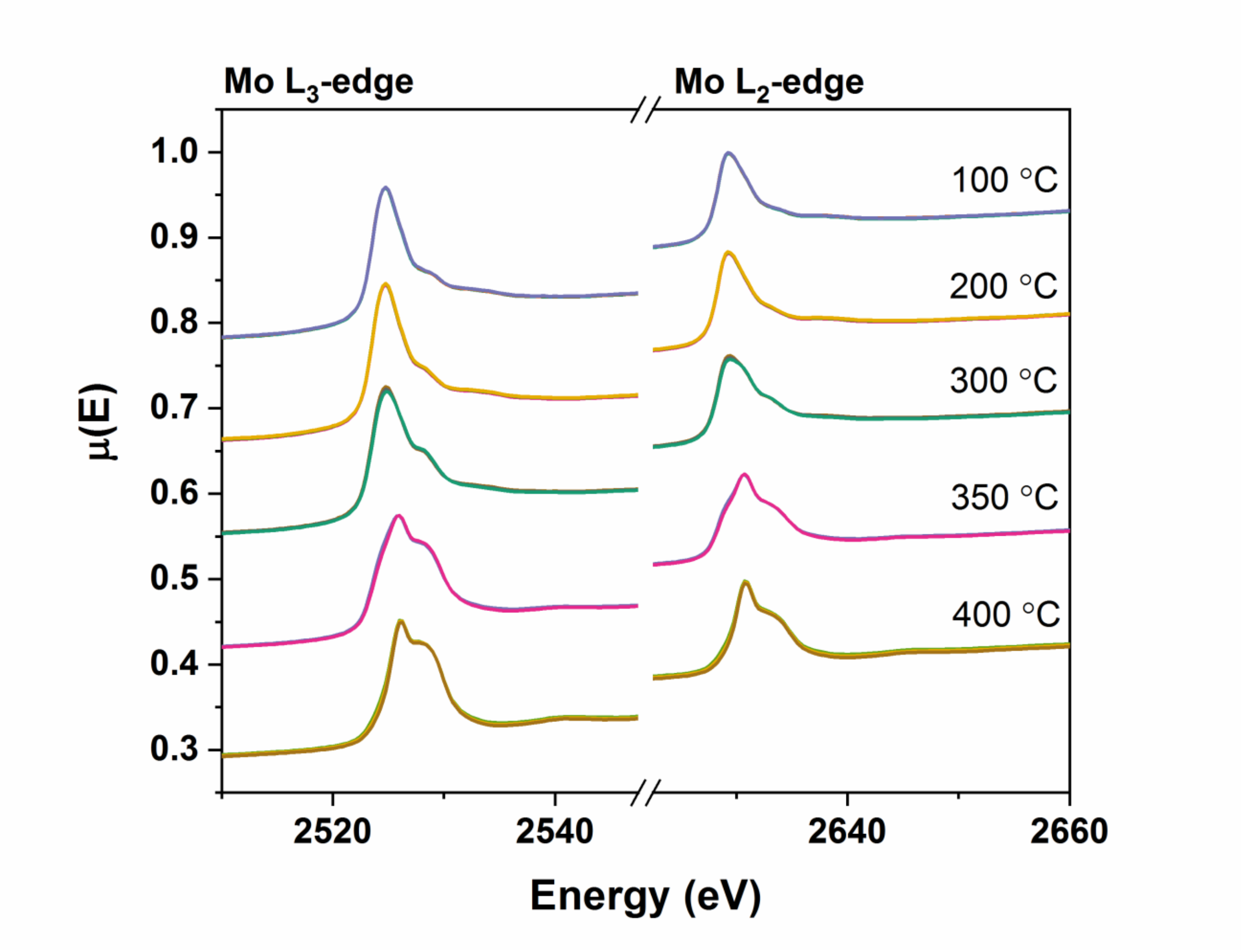

Figure S10. The unprocessed operando Mo $L_{3,2}$-edge X-ray absorption scans of as-grown MoS_2 monolayers. The operando X-ray absorption near edge structure (XANES) of monolayer MoS_2 was obtained with increasing temperature under 3% vol O_2/He atmosphere. The MoS_2 monolayers were ramped to the desired temperature and held there for 30 minutes before recording the three consecutive XAS scans that are shown in the figure (overlapping signals). The second and third scans are performed at 50 min and 70 min of the oxidative heat treatment at the desired temperature, respectively. The spectra in the figure are shown as obtained without normalization, and all the spectra overlap without significant changes indicating steady-state conditions.

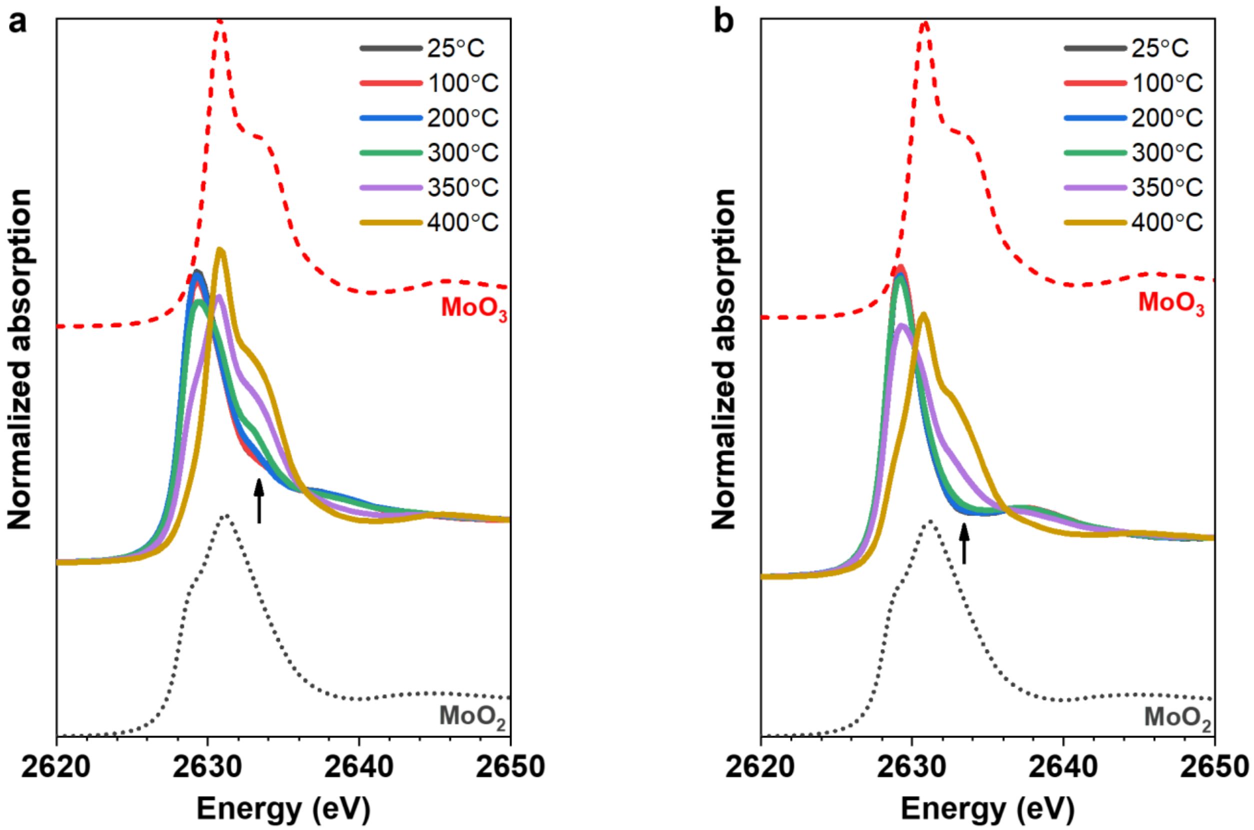

Figure S11. The operando Mo L_2-edge XANES spectra of the as-grown and etched MoS_2 monolayers. a),b) The spectra of the as-grown (a) and etched (b) MoS_2 monolayers were recorded in 3 vol % O_2/He (101.3 kPa) under increasing temperature. The spectra of standard bulk MoO_2 and MoO_3 materials are shown as references with dotted black and dashed red lines, respectively.

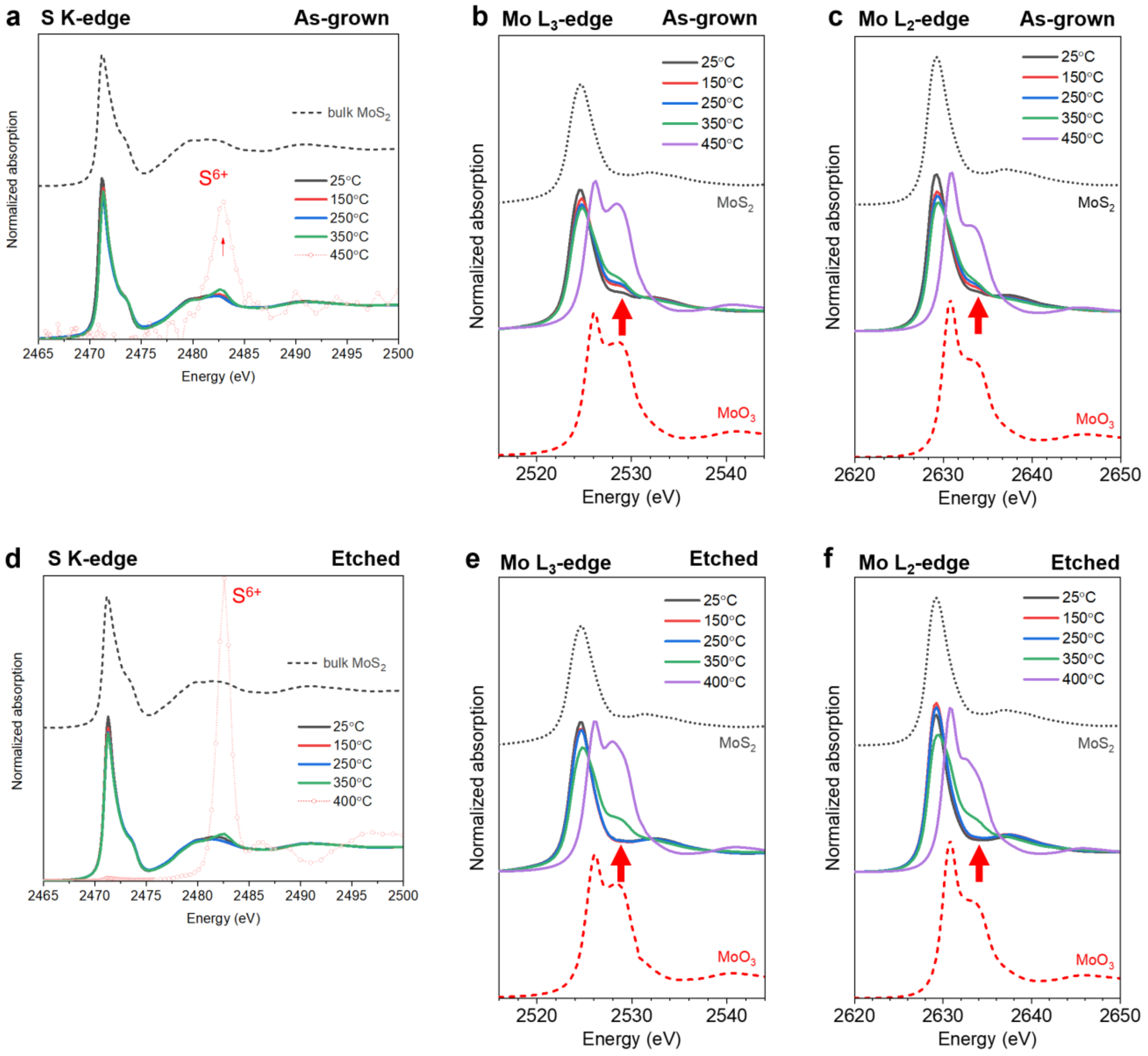

Figure S12. Ex-situ S K-edge, Mo L_3-edge, and Mo L_2-edge XANES spectra obtained after thermal annealing of the as-grown (a-c) and etched (d-f) MoS_2 monolayers under the oxidative atmosphere at different temperatures (3 vol % O_2 in Ar balance, 101.3 kPa). Bulk MoS_2 and MoO_3 standards are shown as references. Red arrows emphasize regions of change where the MoO_x features can be observed in the Mo $L_{2,3}$-edges XANES spectra.

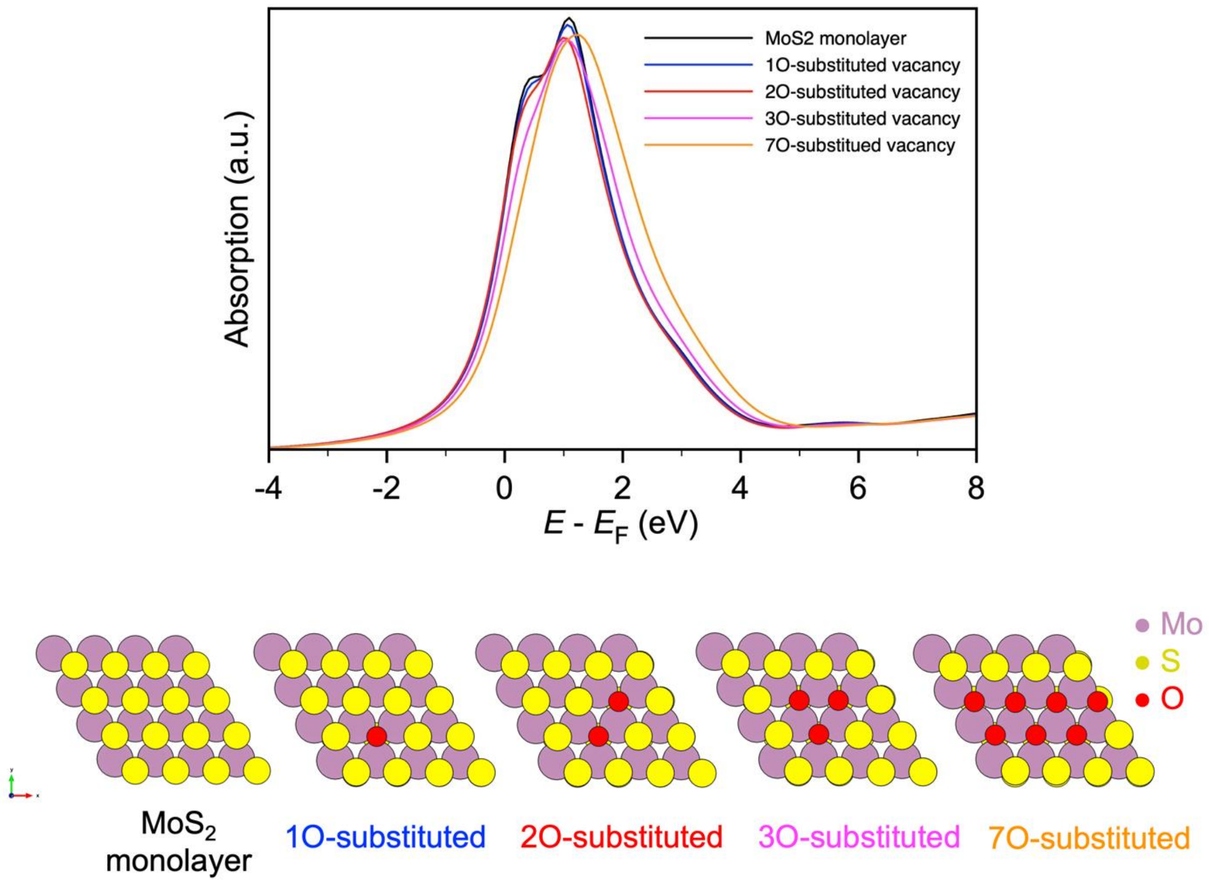

Figure S13. Simulated Mo L_3-edge XANES spectra of MoS_2 monolayer with and without substitution of sulfur atoms by oxygen. First principle calculations were performed with the OCEAN code[12, 13] for a monolayer of pristine MoS_2 and those with S-vacancies that are filled by O atoms (i.e., S atoms are substituted by O). The lower panel shows the structure of the MoS_2 models studied here. Prior to simulating the spectra, all the structures were optimized using density functional theory (DFT) calculations. All atomic coordinates and unit cells were allowed to relax during the optimizations. The theoretical simulations show that O substitutions produce minor effects on the Mo L_3-edge spectra, and therefore O substitution cannot be a major contributor to the spectral differences observed in the experimental results. The most prominent effect of the highest O concentration we simulated is the white line shift to higher energy by ca. 0.18 eV,

without major perturbations to the overall structure); which indicates that the oxidation state of the Mo atom slightly increases relatively to pristine MoS_2. Nonetheless, the main spectral change in our experiment occurs at 2528.6 (≈ 4-5 eV above the absorption edge); which is completely absent from our first-principles simulations, when assuming O substitutions. Hence, the new spectral feature can be confidently assigned to the formation of a new species having the Mo atom in a different chemical environment (mixed phases). This is also supported by the experimental spectra of the reference MoO_2 and MoO_3 standards (Figure S8) and their calculated theoretical spectra showing a spectral peak feature at approximately 3-5 eV above the edge position (Figure S9).

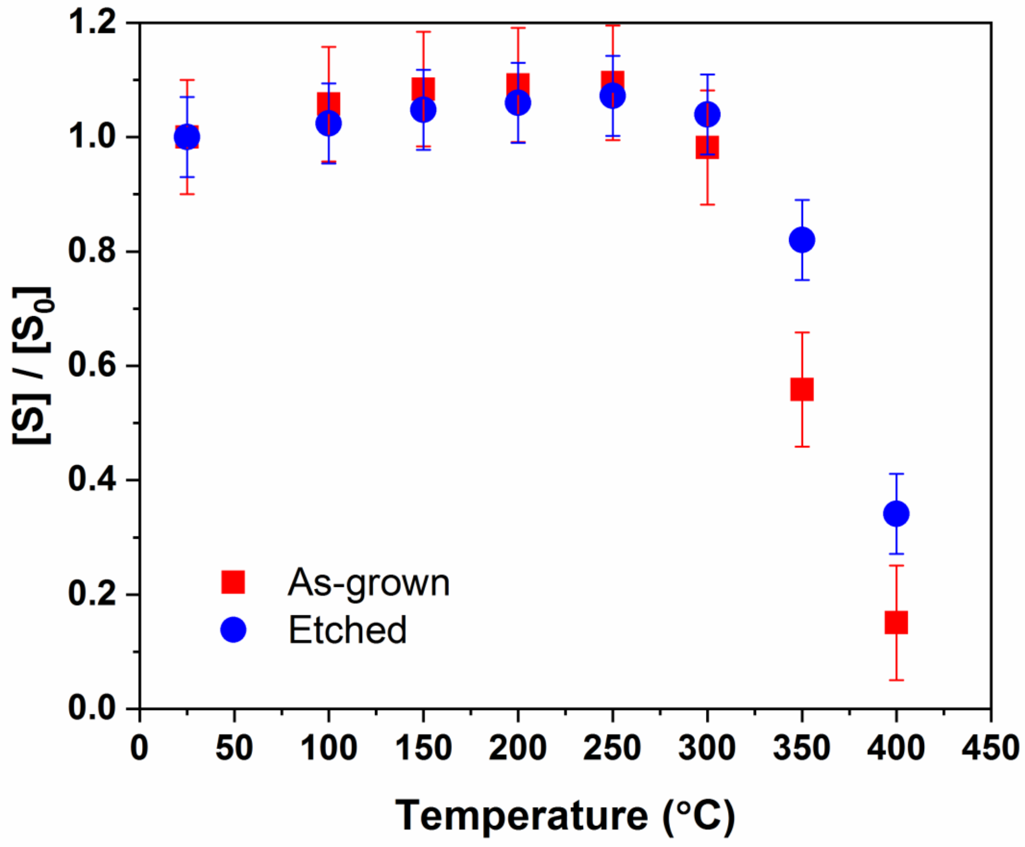

Figure S14. Chemical composition analysis of the as-grown and etched MoS_2 monolayers based on the operando S K-edge XANES spectra. The normalized S K-edge absorption step shows the severe loss of sulfur (S) with increasing oxidation temperatures at 350 °C and 400 °C. The etched MoS_2 monolayers contain ≈ 23 % more sulfur than the as-grown MoS_2 monolayers on average under the same annealing temperatures and oxidation conditions at 350 °C and 400 °C. The error bar is calculated from the standard deviation of the initial sulfur concentration value below 250°C as extracted from the normalization procedure performed with ATHENA (the S amount is normalized to the S K-edge step at room temperature).

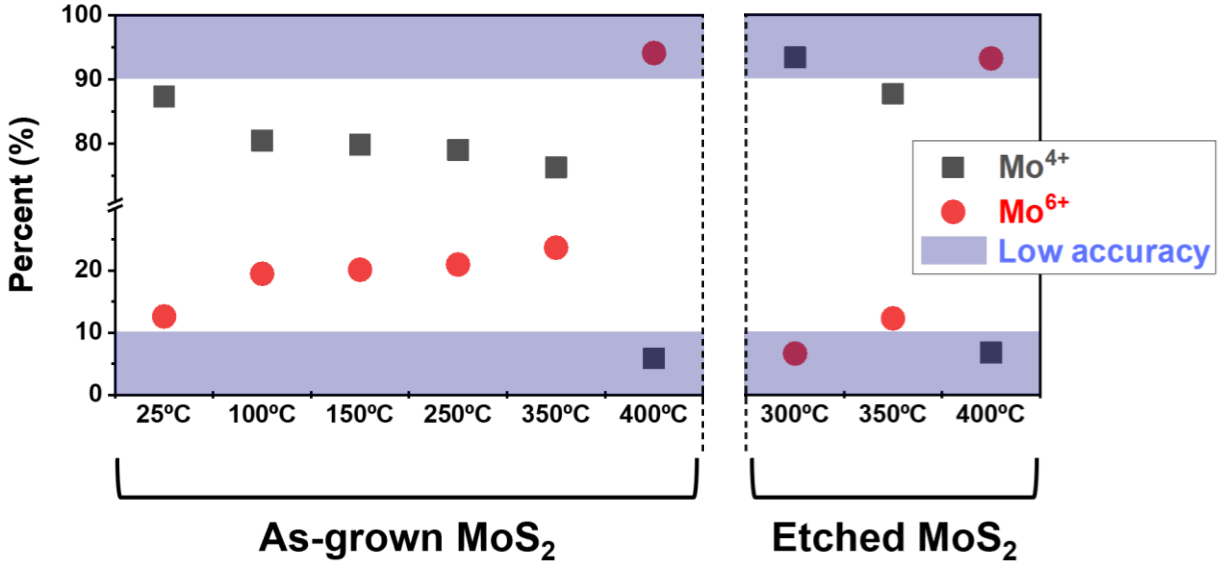

Figure S15. The oxidation state ratio of Mo between Mo^{4+} and Mo^{6+} as obtained from the Mo 3d XPS spectra. The composition was calculated by the peak deconvolution of Figure 4b,e. The high-resolution Mo 3d spectra doublet at 233.3 eV and 230.2 eV were assigned to Mo^{4+} $3d_{3/2}$ and $3d_{5/2}$ respectively. The Mo 3d spectra doublet at 236.5 eV and 233.5 eV were assigned to Mo^{6+} $3d_{3/2}$ and $3d_{5/2}$ respectively in the form of MoO_3.[57-59] The Mo^{6+} peaks increase gradually with increasing temperature in the as-grown monolayer MoS_2 annealed from 100 °C. On the other hand, the etched MoS_2 does not show the distinct Mo^{6+} peak below 300 °C, and the high energy peak at 236.5 eV appeared only when the temperature is equal to and greater than 300 °C. Similarly, for both the as-grown and etched MoS_2 samples, a sudden oxidation transition occurs between 350 °C and 400 °C (Mo^{6+} > 90 %). The annealing conditions are 3 vol % O_2/Ar, ambient pressure, 30 minutes in each denoted temperature. The Mo^{4+} to Mo^{6+} ratios were estimated by fitting the Mo^{4+} and Mo^{6+} 3d peaks (filled color with lines) in Figure 4.

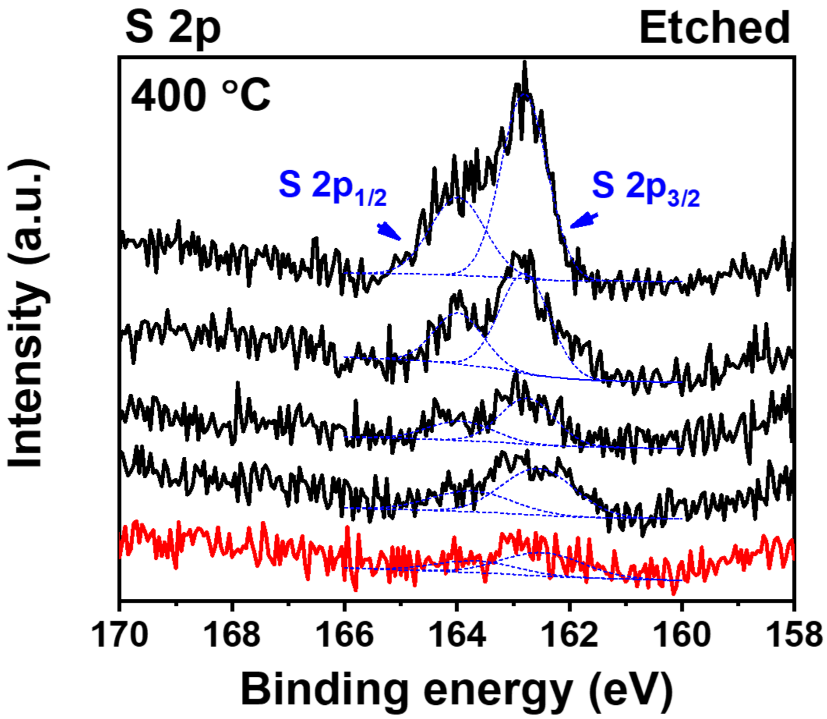

Figure S16. Locational variation of the etched MoS_2 monolayers (400 °C, 30 minutes). XPS spectra measured from the etched MoS_2 monolayers in the same sample but at five different X-ray spots. The full scale of the y-axis of Figure S16 is 10 times magnified compared to that in Figure 4f. The red curve is the same spectrum as the S 2p (400 °C) in Figure 4f, which shows a weak intensity of S 2p in the blue dotted lines for peak deconvolution. The figure shows small intensity variations of the S 2p spectra measured by XPS on varying locations of the sample (PHI Versaprobe 1 with monochromatized Al Kα, X-ray spot size is 250 μm).

Reference	MoS_2 Layers	Environment	Temperature	Time	Characterization
Ref. [60]	1L	Air	380 °C	10 min	OM[a]
Ref. [58]	Few-layer	Air	250 – 310 °C	1 h	XPS[b], AFM[c]
Ref. [61]	Thick flake	Air	360 °C	5 min	AFM, SEM[d]
Ref. [50]	1L, 2L	O_2/Ar	320 °C	1 h	AFM, Raman[e]
Ref. [62]	1 – 5L	H_2O/Ar	500 °C	30 min	AFM
Ref. [63]	2L	Air	330 °C	1h	OM
Ref. [64]	5L	Air	320 °C	5 min	AFM

Table S1. Literature survey of the temperature and time required to observe oxidation features (e.g., cracks, pits, etc.) in a few-layer and monolayer MoS_2 after reaching quasi-equilibrium conditions using different characterization techniques. OM[a]: optical microscopy, XPS[b]: X-ray photoelectron spectroscopy, AFM[c]: atomic force microscopy, SEM[d]: scanning electron microscopy, Raman[e]: Raman spectroscopy

Reference	Material	Method	Detection Mode
This work	Monolayer MoS_2	Operando XAS	Electron yield
Ref. [65]	Amorphous Cobalt	In situ XAS	Fluorescence
Ref. [66]	MoS_2 bulk	Ex-situ XAS	Transmission
Ref. [67]	$Ti_3C_2T_x$ MXene	In situ XAS	Fluorescence
Ref. [68]	NiCo-UMOFNs	In situ and ex situ XAS	Fluorescence, Transmission
Ref. [69]	Co-Fe oxide	Operando HERFD-XAS	Fluorescence
Ref. [70]	Co-Fe-N-C	Operando XAS	Fluorescence
Ref. [71]	NiCoFeP oxyhydroxides	In situ soft XAS	Fluorescence
Ref. [51]	Multilayer $CoMoS_{2-x}$	Ex-situ XAS	Fluorescence
Ref. [72]	CuO_x on Ag nanowire	In situ XAS	Fluorescence
Ref. [73]	Cu	In situ XAS	Fluorescence
Ref. [74]	Fe-N-C	Operando XAS	Fluorescence,
Ref. [75]	P substituted $CoSe_2$	In situ XAS	Fluorescence

Table S2. Summary of comparison among literature studies about in situ and ex situ XAS and this work. Our methodology employs the electron yield detection mode using tender X-rays (2-3 keV) for the characterization of ultra-diluted concentrations such as monolayer MoS_2 as shown in Figure S7. The electron-yield mode is practically based on the Auger electrons, photoelectrons, and secondary electrons overall, that escape from the sample's surface as a function of the energy of the incident X-ray radiation. After X-ray absorption, the electrons escaping the sample's surface ionize the present gas creating multiple electron-hole events which are ultimately events in the environmental gas-phase via impact ionization. Hence, this cascading effect leads to a significantly higher signal (electron yield) than those encountered by the conventional XAS modes.[15, 76-79]

Temperature (ºC)	R-factor	Reduced Chi-squared	MoS_2	MoO_3	MoO_2
		**As-grown MoS_2 monolayers**			
**25**	0.0005	0.001	0.801 (5)	0.134 (8)	0.066 (9)
**100**	0.0009	0.002	0.754 (6)	0.16 (1)	0.08 (1)
**150**	0.002	0.004	0.721 (8)	0.20 (2)	0.08 (1)
**200**	0.001	0.003	0.706 (7)	0.20 (2)	0.09 (1)
**250**	0.001	0.003	0.669 (7)	0.22 (2)	0.11 (1)
**300**	0.001	0.003	0.588 (7)	0.26 (2)	0.16 (1)
**350**	0.0005	0.001	0.308 (6)	0.38 (1)	0.31 (1)
**400**	0.001	0.004	0.03 (1)	0.69 (1)	0.28 (1)
		**Etched MoS_2 monolayers**			
**25**	0.002	0.004	1.0	0	0
**100**	0.0006	0.001	1.0	0	0
**150**	0.001	0.002	1.0	0	0
**200**	0.0009	0.002	1.0	0	0
**250**	0.0004	0.001	1.0	0	0
**300**	0.0003	0.0006	0.945 (5)	0.01 (1)	0.04 (1)
**350**	0.002	0.005	0.652 (9)	0.23 (2)	0.11 (2)
**400**	0.0009	0.003	0.157 (9)	0.43 (1)	0.41 (1)

Table S3. Chemical composition via the linear combination fitting analysis (LCFA) of the operando Mo L_3-edge X-ray absorption near edge structure (XANES) spectra. The fitting of the spectra was done using as reference standards bulk MoO_2 and MoO_3, and the room temperature spectra of the etched monolayer MoS_2 (*i.e.*, pristine monolayer MoS_2 without oxides). The analysis was done with ATHENA software (part of the DEMETER package v0.9.26)[11] within an energy range of −20 eV below to +30 eV above the edge (defined as the first derivative of the Mo L_3-edge white line). LCFA reconstructs the sample spectra using the aforementioned model standards (*i.e.*, etched monolayer MoS_2, MoO_2, and MoO_3). The goodness of fit is reported using the R-factor and the reduced chi-squared. The table below shows the percent that each model contributes to the fit. The relative amounts of MoS_2, MoO_3, and MoO_2 in Figure 3g,h were obtained assuming that all components weights' in the fitting add to unity and constraining the weights of the components as positive values.

	MoS_2 & MoO_3 & MoO_2		MoS_2 & MoO_3		MoS_2 & MoO_2	
Temperature (°C)	R-factor	Reduced Chi-squared	R-factor	Reduced Chi-squared	R-factor	Reduced Chi-squared
As-grown MoS_2 monolayers						
25	**0.0005**	**0.001**	0.0007	0.002	0.002	0.004
100	**0.0009**	**0.002**	0.001	0.003	0.003	0.006
150	**0.002**	**0.004**	0.002	0.004	0.005	0.01
200	**0.001**	**0.003**	0.002	0.004	0.005	0.01
250	**0.001**	**0.003**	0.002	0.005	0.006	0.01
300	**0.001**	**0.003**	0.003	0.006	0.007	0.01
350	**0.0005**	**0.001**	0.004	0.01	0.008	0.02
400	**0.001**	**0.004**	0.006	0.02	0.02	0.07
Etched MoS_2 monolayers						
25	**0.002**	**0.004**	0.002	0.004	0.002	0.004
100	**0.0006**	**0.001**	0.002	0.005	0.003	0.006
150	**0.001**	**0.002**	0.001	0.002	0.001	0.002
200	**0.0009**	**0.002**	0.002	0.004	0.0009	0.002
250	**0.0004**	**0.001**	0.0008	0.002	0.0008	0.002
300	**0.0003**	**0.0006**	0.0004	0.0008	0.0003	0.0006
350	**0.002**	**0.005**	0.003	0.007	0.007	0.02
400	**0.0009**	**0.003**	0.007	0.02	0.01	0.04

Table S4. Statistical parameters obtained from the linear combination fitting analysis (LCFA) of the operando Mo L_3-edge X-ray absorption near edge structure (XANES) spectra when including/excluding different reference standards (MoS_2, MoO_2, MoO_3). The analysis was done with ATHENA software (part of the DEMETER package v0.9.26)[11] within an energy range of −20 eV below to +30 eV above the edge (defined as the first derivative of the Mo L_3-edge white line). LCFA reconstructs the sample spectra using the aforementioned model standards (i.e., etched monolayer MoS_2, MoO_2, and MoO_3). The goodness of fit is reported using the R-factor and the reduced chi-squared.[11] As described by the author of the ATHENA software, here the R-factor is the mean square sum of the misfit at each data point.[11] For the reduced chi-squared, due to the solitary nature of time sequence XAS measurements/scans, ATHENA assumes 1 as the value of

measurement uncertainty, resulting in very small values for chi-squared. Hence, although the single values cannot assert the goodness of the fit, it is possible to relatively compare successive fits as provided below. Using all three reference standards results in improvements of a factor of 2 and 5 for the average R-factor for all temperature points for both samples; and, an improvement on the fit by a factor of 2.5 and 5.5 for the average reduced chi-squared values when only considering MoO_3 and MoO_2 standards in the fit, respectively. The fits were performed assuming that all components weights' in the fitting add to unity and constraining the weights of the components as positive values.

References

[1] H. Li, C. Tsai, A. L. Koh, L. Cai, A. W. Contryman, A. H. Fragapane, J. Zhao, H. S. Han, H. C. Manoharan, F. Abild-Pedersen, J. K. Norskov, X. Zheng, *Nat. Mater.* **2016**, *15*, 48.

[2] C. Tsai, H. Li, S. Park, J. Park, H. S. Han, J. K. Norskov, X. Zheng, F. Abild-Pedersen, *Nat. Commun.* **2017**, *8*, 15113.

[3] H. Li, Q. Zhang, C. C. R. Yap, B. K. Tay, T. H. T. Edwin, A. Olivier, D. Baillargeat, *Adv. Funct. Mater.* **2012**, *22*, 1385.

[4] J. Miao, F.-X. Xiao, H. B. Yang, S. Y. Khoo, J. Chen, Z. Fan, Y.-Y. Hsu, H. M. Chen, H. Zhang, B. Liu, *Sci. Adv.* **2015**, *1*, e1500259.

[5] I. Song, C. Park, M. Hong, J. Baik, H. J. Shin, H. C. Choi, *Angew. Chem.-Int. Edit.* **2014**, *53*, 1266.

[6] V. K. Sangwan, H. S. Lee, H. Bergeron, I. Balla, M. E. Beck, K. S. Chen, M. C. Hersam, *Nature* **2018**, *554*, 500.

[7] X. Zhang, J. Grajal, J. L. Vazquez-Roy, U. Radhakrishna, X. Wang, W. Chern, L. Zhou, Y. Lin, P. C. Shen, X. Ji, X. Ling, A. Zubair, Y. Zhang, H. Wang, M. Dubey, J. Kong, M. Dresselhaus, T. Palacios, *Nature* **2019**, *566*, 368.

[8] A. G. Tyurin, *Protect. Met.* **2003**, *39*, 367.

[9] J. Zhu, H. Xu, G. Zou, W. Zhang, R. Chai, J. Choi, J. Wu, H. Liu, G. Shen, H. Fan, *J. Am. Chem. Soc.* **2019**, *141*, 5392.

[10] A. Azcatl, S. McDonnell, S. K. C, X. Peng, H. Dong, X. Qin, R. Addou, G. I. Mordi, N. Lu, J. Kim, M. J. Kim, K. Cho, R. M. Wallace, *Appl. Phys. Lett.* **2014**, *104*.

[11] B. Ravel, M. Newville, *J. Synchrot. Radiat.* **2005**, *12*, 537.

[12] J. Vinson, J. J. Rehr, J. J. Kas, E. L. Shirley, *Phys. Rev. B-Condens Matter* **2011**, *83*.

[13] K. Gilmore, J. Vinson, E. L. Shirley, D. Prendergast, C. D. Pemmaraju, J. J. Kas, F. D. Vila, J. J. Rehr, *Comput. Phys. Commun.* **2015**, *197*, 109.

[14] P. Giannozzi, S. Baroni, N. Bonini, M. Calandra, R. Car, C. Cavazzoni, D. Ceresoli, G. L. Chiarotti, M. Cococcioni, I. Dabo, A. Dal Corso, S. de Gironcoli, S. Fabris, G. Fratesi, R. Gebauer, U. Gerstmann, C. Gougoussis, A. Kokalj, M. Lazzeri, L. Martin-Samos, N. Marzari, F. Mauri, R. Mazzarello, S. Paolini, A. Pasquarello, L. Paulatto, C. Sbraccia, S.

Scandolo, G. Sclauzero, A. P. Seitsonen, A. Smogunov, P. Umari, R. M. Wentzcovitch, *J. Phys.-Condes. Matter* **2009**, *21*, 395502.

[15] W. T. Elam, J. P. Kirkland, R. A. Neiser, P. D. Wolf, *Phys. Rev. B-Condens Matter* **1988**, *38*, 26.

[16] K. F. Garrity, J. W. Bennett, K. M. Rabe, D. Vanderbilt, *Comput. Mater. Sci.* **2014**, *81*, 446.

[17] B. Hammer, L. B. Hansen, J. K. Nørskov, *Phys. Rev. B* **1999**, *59*, 7413.

[18] W. Kohn, L. J. Sham, *Phys. Rev.* **1965**, *140*, A1133.

[19] C. P. Schwartz, F. Ponce, S. Friedrich, S. P. Cramer, J. Vinson, D. Prendergast, *J. Electron Spectrosc. Relat. Phenom.* **2017**, *218,* 30.

[20] E. L. Shirley, *Phys. Rev. B* **1996**, *54*, 16464.

[21] J. P. Perdew, K. Burke, M. Ernzerhof, *Phys. Rev. Lett.* **1996**, *77*, 3865.

[22] E. L. Shirley, *Ultramicroscopy* **2006**, *106*, 986.

[23] S. B. Desai, S. R. Madhvapathy, M. Amani, D. Kiriya, M. Hettick, M. Tosun, Y. Zhou, M. Dubey, J. W. Ager, 3rd, D. Chrzan, A. Javey, *Adv. Mater.* **2016**, *28*, 4053.

[24] C. Zhang, Z. Wang, S. Bhoyate, T. Morey, B. Neria, V. Vasiraju, G. Gupta, S. Palchoudhury, P. Kahol, S. Mishra, F. Perez, R. Gupta, *C* **2017**, *3*.

[25] H. Li, A. W. Contryman, X. Qian, S. M. Ardakani, Y. Gong, X. Wang, J. M. Weisse, C. H. Lee, J. Zhao, P. M. Ajayan, J. Li, H. C. Manoharan, X. Zheng, *Nat. Commun.* **2015**, *6*, 7381.

[26] X. Li, W. Cai, J. An, S. Kim, J. Nah, D. Yang, R. Piner, A. Velamakanni, I. Jung, E. Tutuc, S. K. Banerjee, L. Colombo, R. S. Ruoff, *Science* **2009**, *324*, 1312.

[27] K. S. Kim, Y. Zhao, H. Jang, S. Y. Lee, J. M. Kim, K. S. Kim, J. H. Ahn, P. Kim, J. Y. Choi, B. H. Hong, *Nature* **2009**, *457*, 706.

[28] L. Benoist, D. Gonbeau, G. Pfister-Guillouzo, E. Schmidt, G. Meunier, A. Levasseur, *Surf. Interface Anal.* **1994**, *22*, 206.

[29] J. C. Muijsers, T. V. Weber, R. M. anhardeveld, H. W. Zandbergen, J. W. Niemantsverdriet, *J. Catal.* **1995**, *157*, 698.

[30] L. Benoist, D. Gonbeau, G. Pfister-Guillouzo, E. Schmidt, G. Meunier, A. Levasseur, *Thin Solid Films* **1995**, *258*, 110.

[31] J. C. Dupina, D. Gonbeaua, I. Martin-Litasb, P. Vinatierb, A. Levasseur, *Appl. Surf. Sci.* **2001**, *173*, 140.

[32] X.-Q. Bao, D. Y. Petrovykh, P. Alpuim, D. G. Stroppa, N. Guldris, H. Fonseca, M. Costa, J. Gaspar, C. Jin, L. Liu, *Nano Energy* **2015**, *16*, 130.

[33] P. Cao, J. Peng, S. Liu, Y. Cui, Y. Hu, B. Chen, J. Li, M. Zhai, *Sci Rep* **2017**, *7*, 16048.

[34] S. Najmaei, Z. Liu, W. Zhou, X. Zou, G. Shi, S. Lei, B. I. Yakobson, J. C. Idrobo, P. M. Ajayan, J. Lou, *Nat. Mater.* **2013**, *12*, 754.

[35] S. Li, Y. C. Lin, X. Y. Liu, Z. Hu, J. Wu, H. Nakajima, S. Liu, T. Okazaki, W. Chen, T. Minari, Y. Sakuma, K. Tsukagoshi, K. Suenaga, T. Taniguchi, M. Osada, *Nanoscale* **2019**, *11*, 16122.

[36] B. Chen, Q. Yu, Q. Yang, P. Bao, W. Zhang, L. Lou, W. Zhu, G. Wang, *RSC Adv.* **2016**, *6*, 50306.

[37] I. Delac Marion, D. Capeta, B. Pielic, F. Faraguna, A. Gallardo, P. Pou, B. Biel, N. Vujicic, M. Kralj, *Nanotechnology* **2018**, *29*, 305703.

[38] C.-C. Cheng, A.-Y. Lu, C.-C. Tseng, X. Yang, M. N. Hedhili, M.-C. Chen, K.-H. Wei, L.-J. Li, *Nano Energy* **2016**, *30*, 846.

[39] J. Hong, Z. Hu, M. Probert, K. Li, D. Lv, X. Yang, L. Gu, N. Mao, Q. Feng, L. Xie, J. Zhang, D. Wu, Z. Zhang, C. Jin, W. Ji, X. Zhang, J. Yuan, Z. Zhang, *Nat. Commun.* **2015**, *6*, 6293.

[40] H. Qiu, T. Xu, Z. Wang, W. Ren, H. Nan, Z. Ni, Q. Chen, S. Yuan, F. Miao, F. Song, G. Long, Y. Shi, L. Sun, J. Wang, X. Wang, *Nat. Commun.* **2013**, *4*, 2642.

[41] P. Vancso, G. Z. Magda, J. Peto, J. Y. Noh, Y. S. Kim, C. Hwang, L. P. Biro, L. Tapaszto, *Sci Rep* **2016**, *6*, 29726.

[42] X. Zhang, H. Nan, S. Xiao, X. Wan, Z. Ni, X. Gu, K. Ostrikov, *ACS Appl. Mater. Interfaces* **2017**, *9*, 42121.

[43] J. Chen, W. Tang, B. Tian, B. Liu, X. Zhao, Y. Liu, T. Ren, W. Liu, D. Geng, H. Y. Jeong, H. S. Shin, W. Zhou, K. P. Loh, *Adv. Sci.* **2016**, *3*, 1500033.

[44] J. Lee, S. Pak, P. Giraud, Y. W. Lee, Y. Cho, J. Hong, A. R. Jang, H. S. Chung, W. K. Hong, H. Y. Jeong, H. S. Shin, L. G. Occhipinti, S. M. Morris, S. Cha, J. I. Sohn, J. M. Kim, *Adv. Mater.* **2017**, *29*.

[45] J. Zhang, H. Yu, W. Chen, X. Tian, D. Liu, M. Cheng, G. Xie, W. Yang, R. Yang, X. Bai, D. Shi, G. Zhang, *ACS Nano* **2014**, *8*, 6024.

[46] S. Wang, M. Pacios, H. Bhaskaran, J. H. Warner, *Nanotechnology* **2016**, *27*, 085604.

[47] Y. Yu, C. Li, Y. Liu, L. Su, Y. Zhang, L. Cao, *Sci Rep* **2013**, *3*, 1866.

[48] H. Yu, M. Liao, W. Zhao, G. Liu, X. J. Zhou, Z. Wei, X. Xu, K. Liu, Z. Hu, K. Deng, S. Zhou, J. A. Shi, L. Gu, C. Shen, T. Zhang, L. Du, L. Xie, J. Zhu, W. Chen, R. Yang, D. Shi, G. Zhang, *ACS Nano* **2017**, *11*, 12001.

[49] R. Kappera, D. Voiry, S. E. Yalcin, B. Branch, G. Gupta, A. D. Mohite, M. Chhowalla, *Nat. Mater.* **2014**, *13*, 1128.

[50] M. Yamamoto, T. L. Einstein, M. S. Fuhrer, W. G. Cullen, *J. Phys. Chem. C* **2013**, *117*, 25643.

[51] S. Park, J. Park, H. Abroshan, L. Zhang, J. K. Kim, J. Zhang, J. Guo, S. Siahrostami, X. Zheng, *ACS Energy Lett.* **2018**, *3*, 2685.

[52] L. Zhang, D. Sun, J. Kang, J. Feng, H. A. Bechtel, L. W. Wang, E. J. Cairns, J. Guo, *Nano Lett.* **2018**, *18*, 1466.

[53] F. Cesano, S. Bertarione, A. Piovano, G. Agostini, M. M. Rahman, E. Groppo, F. Bonino, D. Scarano, C. Lamberti, S. Bordiga, L. Montanari, L. Bonoldi, R. Millini, A. Zecchina, *Catal. Sci. Technol.* **2011**, *1*, 123.

[54] H. Zhang, H. Lin, Y. Zheng, *Catalysts* **2018**, *8*.

[55] D. Hara, H. Ikuta, Y. Uchimoto, M. Wakihara, *J. Mater. Chem.* **2002**, *12*, 2507.

[56] H. M. Tsai, K. Asokan, C. W. Pao, J. W. Chiou, C. H. Du, W. F. Pong, M. H. Tsai, L. Y. Jang, *Appl. Phys. Lett.* **2007**, *91*.

[57] J. Kibsgaard, Z. Chen, B. N. Reinecke, T. F. Jaramillo, *Nat. Mater.* **2012**, *11*, 963.

[58] D. M. Sim, M. Kim, S. Yim, M.-J. Choi, J. Choi, S. Yoo, Y. S. Jung, *ACS Nano* **2015**, *9*, 12115.

[59] J. Gao, B. Li, J. Tan, P. Chow, T. M. Lu, N. Koratkar, *ACS Nano* **2016**, *10*, 2628.

[60] E. Yalon, C. J. McClellan, K. K. H. Smithe, M. Munoz Rojo, R. L. Xu, S. V. Suryavanshi, A. J. Gabourie, C. M. Neumann, F. Xiong, A. B. Farimani, E. Pop, *Nano Lett.* **2017**, *17*, 3429.

[61] W. L. Spychalski, M. Pisarek, R. Szoszkiewicz, *J. Phys. Chem. C* **2017**, *121*, 26027.

[62] Z. Wang, Q. Li, H. Xu, C. Dahl-Petersen, Q. Yang, D. Cheng, D. Cao, F. Besenbacher, J. V. Lauritsen, S. Helveg, M. Dong, *Nano Energy* **2018**, *49*, 634.

[63] J. Wu, H. Li, Z. Yin, H. Li, J. Liu, X. Cao, Q. Zhang, H. Zhang, *Small* **2013**, *9*, 3314.

[64] U. Ukegbu, R. Szoszkiewicz, *J. Phys. Chem. C* **2019**, *123*, 22123.

[65] B. Lassalle-Kaiser, A. Zitolo, E. Fonda, M. Robert, E. Anxolabéhère-Mallart, *ACS Energy Lett.* **2017**, *2*, 2545.

[66] C. D. Quilty, L. M. Housel, D. C. Bock, M. R. Dunkin, L. Wang, D. M. Lutz, A. Abraham, A. M. Bruck, E. S. Takeuchi, K. J. Takeuchi, A. C. Marschilok, *ACS Appl. Energy Mater.* **2019**, *2*, 7635.

[67] M. R. Lukatskaya, S.-M. Bak, X. Yu, X.-Q. Yang, M. W. Barsoum, Y. Gogotsi, *Adv. Energy Mater.* **2015**, *5*.

[68] S. Zhao, Y. Wang, J. Dong, C.-T. He, H. Yin, P. An, K. Zhao, X. Zhang, C. Gao, L. Zhang, J. Lv, J. Wang, J. Zhang, A. M. Khattak, N. A. Khan, Z. Wei, J. Zhang, S. Liu, H. Zhao, Z. Tang, *Nat. Energy* **2016**, *1*.

[69] S. F. Hung, Y. T. Chan, C. C. Chang, M. K. Tsai, Y. F. Liao, N. Hiraoka, C. S. Hsu, H. M. Chen, *J. Am. Chem. Soc.* **2018**, *140*, 17263.

[70] L. Bai, C. S. Hsu, D. T. L. Alexander, H. M. Chen, X. Hu, *J. Am. Chem. Soc.* **2019**, *141*, 14190.

[71] X. Zheng, B. Zhang, P. De Luna, Y. Liang, R. Comin, O. Voznyy, L. Han, F. P. Garcia de Arquer, M. Liu, C. T. Dinh, T. Regier, J. J. Dynes, S. He, H. L. Xin, H. Peng, D. Prendergast, X. Du, E. H. Sargent, *Nat. Chem.* **2018**, *10*, 149.

[72] C. J. Chang, S. F. Hung, C. S. Hsu, H. C. Chen, S. C. Lin, Y. F. Liao, H. M. Chen, *ACS Central Sci.* **2019**, *5*, 1998.

[73] P. De Luna, R. Quintero-Bermudez, C.-T. Dinh, M. B. Ross, O. S. Bushuyev, P. Todorović, T. Regier, S. O. Kelley, P. Yang, E. H. Sargent, *Nat. Catal.* **2018**, *1*, 103.

[74] J. Gu, C. S. Hsu, L. Bai, H. M. Chen, X. Hu, *Science* **2019**, *364*, 1091.

[75] Y. Zhu, H.-C. Chen, C.-S. Hsu, T.-S. Lin, C.-J. Chang, S.-C. Chang, L.-D. Tsai, H. M. Chen, *ACS Energy Lett.* **2019**, *4*, 987.

[76] N. J. Shevchik, D. A. Fischer, *Rev. Sci. Instrum.* **1979**, *50*, 577.

[77] C. E. Bouldin, R. A. Forman, M. I. Bell, *Phys. Rev. B-Condens Matter* **1987**, *35*, 1429.

[78] A. Erbil, I. G. Cargill, R. Frahm, R. F. Boehme, *Phys. Rev. B-Condens Matter* **1988**, *37*, 2450.

[79] G. D. Moggridge, T. Rayment, R. M. Ormerod, M. A. Morris, R. M. Lambert, *Nature* **1992**, *358*, 658.
